# Novel full-thickness biomimetic corneal model for studying pathogenesis and treatment of diabetic keratopathy

**DOI:** 10.1016/j.mtbio.2024.101409

**Published:** 2024-12-16

**Authors:** Zekai Cui, Xiaoxue Li, Yiwen Ou, Xihao Sun, Jianing Gu, Chengcheng Ding, Zhexiong Yu, Yonglong Guo, Yuqin Liang, Shengru Mao, Jacey Hongjie Ma, Hon Fai Chan, Shibo Tang, Jiansu Chen

**Affiliations:** a Aier Academy of Ophthalmology, Central South University, Changsha, Hunan, China; b Changsha Aier Eye Hospital, Changsha, Hunan, China; c Tianren Goji Biotechnology Co., Ltd, Ningxia, China; d College of Veterinary Medicine, South China Agricultural University, Guangzhou, China; e Institute for Tissue Engineering and Regenerative Medicine, The Chinese University of Hong Kong, Hong Kong, China

**Keywords:** Diabetic keratopathy (DK), Biomimetic full-thickness corneal model, Lycium barbarum glycopeptide (LBGP), C-C motif chemokine ligand 5 (CCL5)

## Abstract

Diabetic keratopathy (DK), a significant complication of diabetes, often leads to corneal damage and vision impairment. Effective models are essential for studying DK pathogenesis and evaluating potential therapeutic interventions. This study developed a novel biomimetic full-thickness corneal model for the first time, incorporating corneal epithelial cells, stromal cells, endothelial cells, and nerves to simulate DK conditions *in vitro*. By exposing the model to a high-glucose (HG) environment, the pathological characteristics of DK, including nerve bundle disintegration, compromised barrier integrity, increased inflammation, and oxidative stress, were successfully replicated. Transcriptomic analysis revealed that HG downregulated genes associated with axon and synapse formation while upregulating immune response and oxidative stress pathways, with C-C Motif Chemokine Ligand 5 (CCL5) identified as a key hub gene in DK pathogenesis. The therapeutic effects of Lycium barbarum glycopeptide (LBGP) were evaluated using this model and validated in db/db diabetic mice. LBGP promoted nerve regeneration, alleviated inflammation and oxidative stress in both *in vitro* and *in vivo* models. Notably, LBGP suppressed the expression of CCL5, highlighting its potential mechanism of action. This study establishes a robust biomimetic platform for investigating DK and other corneal diseases, and identifies LBGP as a promising therapeutic candidate for DK. These findings provide valuable insights into corneal disease mechanisms and pave the way for future translational research and clinical applications.

## Highlights

• A full-thickness biomimetic corneal model containing corneal epithelium, nerves, stroma, and endothelium was constructed.
• Using this model, the pathological characteristics of diabetic keratopathy were successfully replicated *in vitro*.
• Lycium barbarum glycopeptide (LBGP) alleviated high-glucose-induced damage *in vitro* and *in vivo* models.
• CCL5 plays an important role in the pathogenesis of diabetic keratopathy.

## Graphical abstract

**Figure:**
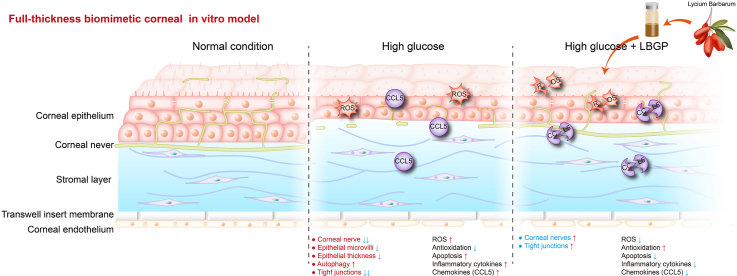

## 1 Introduction

Diabetic keratopathy (DK) is a degenerative corneal disease commonly observed in patients with systemic diabetes mellitus. In 2017, the global prevalence of diabetes among adults was approximately 451 million, projected to rise to 693 million by 2045 [1]. Approximately 46–64 % of diabetic patients develop diabetic keratopathy during their disease [2]. The main clinical manifestations of DK include reduced corneal nerve density and sensitivity, epithelial defects, delayed wound healing, corneal erosion, decreased corneal cell density, increased corneal thickness, and altered corneal biomechanical properties [3]. These changes significantly impair corneal physiological function, leading to substantial vision loss and even blindness. The pathogenesis of DK involves multiple complex factors, including hyperglycemia-induced oxidative stress, the polyol pathway, non-enzymatic glycation of proteins, and the accumulation of advanced glycation end-products (AGEs). These factors contribute to corneal cell damage through increased production of reactive oxygen species (ROS) [4]. Hyperglycemia also leads to corneal nerve degeneration and reduced nerve growth factor (NGF) levels, impairing corneal sensation and repair capacity [5]. Furthermore, metabolic dysregulation and the release of inflammatory cytokines (such as IL-1, IL-6, and TNF-α) activate the NF-κB pathway, triggering inflammation and cellular damage [4]. Signaling pathways such as ERK [6], PI3K/Akt [7], and TGF-β [8] are abnormally activated under hyperglycemia and oxidative stress, disrupting corneal epithelial cell function and repair, promoting fibrosis, and remodeling the extracellular matrix. These mechanisms interact synergistically, contributing to the onset and progression of diabetic keratopathy. Current clinical treatment strategies for DK include strict glycemic control, local ocular treatments, surgical interventions, and emerging therapies. Maintaining optimal blood glucose levels is fundamental in managing DK and can be achieved through a balanced diet, insulin therapy, or oral hypoglycemic agents such as metformin and sulfonylureas [5]. Local ocular treatments include artificial tears to alleviate symptoms of dry eye, and antibiotic eye drops such as levofloxacin to prevent and treat corneal infections, corneal protectants like vitamin A eye drops to promote epithelial healing, and corticosteroid eye drops used cautiously to reduce inflammation. Surgical interventions are also utilized, including corneal cross-linking to enhance corneal stromal stability and corneal transplantation to replace damaged tissue in severe cases [9]. Neuroprotective agents, such as nerve growth factor (NGF), help regenerate corneal nerve fibers and promote epithelial healing [10]. Emerging therapies like gene therapy and stem cell therapy are currently under investigation and hold promise for the future treatment of DK. Despite these available treatments, there is still a lack of specific drugs that effectively target the underlying mechanisms of diabetic keratopathy, and necessitating continued research and the exploration of new therapeutic strategies to improve the management and outcomes of DK.

In biomedical research, *in vitro*, *in vivo*, and mathematical models play distinct but complementary roles in advancing our understanding of biological systems and disease mechanisms. *In vitro* models offer controlled environments to study specific cellular and molecular interactions, enabling precise manipulation of variables such as drug concentrations or environmental conditions. These models are ideal for early-stage research and high-throughput testing [11,12]. *In vivo* models, on the other hand, provide a more comprehensive understanding by capturing the complexity of systemic interactions within a living organism, making them crucial for validating findings and assessing physiological relevance [12]. Complementing these experimental approaches, mathematical models are powerful tools for simulating complex biological processes, predicting outcomes, and integrating data from both *in vitro* and *in vivo* studies. For example, they can be used to mathematically model disease transmission [13,14], tissue biomechanics [15], or neural networks [16], thereby reducing reliance on animal experiments and optimizing experimental design. Together, these models form an interconnected framework, where findings from one type can inform and refine the others, enabling a deeper understanding of biological systems and accelerating the development of therapeutic strategies.

*In vitro* models offer numerous significant advantages in developing corneal drugs and studying corneal diseases. Firstly, *in vitro* models reduce the reliance on animal experiments, adhering to ethical standards and the 3Rs principles (Replacement, Reduction, and Refinement). Moreover, compared to animal experiments, using *in vitro* models offers several advantages, including simpler and faster molecular and genetic manipulation, reduced experimental time, lower costs, and more reproducible results [17]. Ranging from simple 2D monolayer cell cultures to complex 3D corneal models, *in vitro* systems offer a variety of research tools to meet different research needs. Most importantly, these models enable studying cell behavior and response under specific conditions, providing crucial information for understanding drug mechanisms of action [18]. Researchers have recently developed various *in vitro* corneal models to study the interactions between corneal cells and nerves in physiological and pathological processes. Sharif et al. developed a co-culture model of corneal stromal cells and nerves using transwell systems, which is simple to operate and valuable for drug screening [19]. Wang et al. constructed a 3D corneal model using silk films, incorporating multiple stromal layers, epithelial layers, and sensory nerves [20]. Their colleagues used this 3D *in vitro* corneal tissue model to study the transient receptor potential (TRP) channel vanilloid 1 and anchor protein 1 (TRPV1; TRPA1) in response to allyl isothiocyanate (AITC) stimulation [21]. Our team previously developed a corneal stroma-nerve co-culture model, identifying crucial mechanisms promoting nerve growth and screening neurotrophic drugs using this model [22]. Additionally, some models have been designed to simulate corneal cell pathology in DK. For instance, studies have utilized corneal fibroblasts derived from diabetic patients to self-assemble into 3D corneal stroma, exploring pathological changes [23]. Priyadarsini et al. self-assembled corneal stromal layers from corneal fibroblasts of both healthy individuals and diabetic patients, assessing the morphological and molecular biological differences in co-culture systems with nerve cells [24]. The silk film co-culture system mentioned earlier also showed changes in neuronal cell morphology under high-glucose conditions [25]. However, there have been no reports of *in vitro* models that comprehensively simulate the histological and molecular biological characteristics of DK. Therefore, establishing a biomimetic full-thickness *in vitro* corneal model comprising epithelial, nerve, stromal, and endothelial components is paramount for studying DK's pathological mechanisms and screening effective therapeutic drugs.

Lycium barbarum, commonly known as goji berry, is a traditional Chinese medicinal herb renowned for its anti-aging, vision-improving, and anti-fatigue properties [26]. The beneficial effects of Lycium barbarum extract on ocular diseases have been extensively reported, including improvements in conditions such as glaucoma, retinal photodamage, retinitis pigmentosa (RP), and diabetic retinopathy [27]. Lycium barbarum glycopeptide (LBGP), an immunoactive glycoprotein further purified and isolated from Lycium barbarum polysaccharides (LBP) [28]. Studies have demonstrated that LBGP possesses several protective properties across various diseases. LBGP can effectively scavenge free radicals and inhibit the release of inflammatory mediators, thereby reducing oxidative stress and inflammation. It has also been shown to regulate cell apoptosis pathways, offering protection against cell death in various disease models. For instance, LBGP demonstrated protective effects in spinal cord injury by modulating inflammation and promoting neural repair [28], protected retinal cells and improved visual function in models of retinitis pigmentosa [27], alleviated symptoms of anxiety [29], exhibited anti-inflammatory effects in acute colitis models [30], and showed anti-tumor activity in glioblastoma [31]. Despite these promising findings, the potential of LBGP in alleviating the symptoms of DK remains to be explored. Further research is necessary to evaluate whether LBGP can mitigate the pathological changes associated with DK and improve corneal health in diabetic patients, potentially leading to the development of novel therapeutic strategies for managing this challenging condition.

In this study, a biomimetic full-thickness *in vitro* corneal model was first constructed, comprising epithelial cells, nerves, stromal cells, and endothelial cells, and successfully induced into a diabetic keratopathy (DK) model under a high-glucose (HG) environment. Morphological assessments of the normal control (NC) and HG groups were then conducted using transmission electron microscopy (TEM), scanning electron microscopy (SEM), and immunofluorescence staining, with cytokine expression differences analyzed via cytokine microarrays and ELISA. RNA sequencing (RNA-Seq) enabled transcriptomic analysis of each cell type in the NC and HG groups, predicting key genes and signaling pathways. Next, the reparative effects of Lycium barbarum glycopeptide (LBGP) on HG-induced damage were evaluated using the *in vitro* model, focusing on nerve growth, oxidative stress, inflammation, apoptosis, and changes in the expression of key proteins and genes. Finally, the therapeutic effects of LBGP on corneal lesions were further validated using the db/db diabetic mouse model. This study demonstrates the utility of the biomimetic full-thickness *in vitro* corneal model and diabetic mouse model for assessing the therapeutic potential of LBGP, offering valuable insights into *in vitro* corneal model research and innovative treatments for DK.

## 2 Materials and methods

### 2.1 Ethics statement

The use of lenticule tissue samples obtained from Small Incision Lenticule Extraction (SMILE) surgery was approved by the Ethics Committee of Aier Eye Hospital, Changsha, Hunan, China (No. 2023-KYPJ009), and adhered to the principles of the Declaration of Helsinki. Informed consent was obtained from all patients. All animal experiments were approved by the Ethics Committee of the Animal Center of Aier Eye Research Institute (No. AEI20230015) and complied with the guidelines of the National Institutes of Health (NIH) for the care and use of animals, as well as the ARVO Statement for the Use of Animals in Ophthalmic and Vision Research.

### 2.2 Cell culture

Following SMILE, stromal lenticules were collected and stored in DMEM. The lenticules were then digested overnight at 37 °C in DMEM/F12 medium containing 0.1 % Type I collagenase (Sigma-Aldrich, USA). The next day, the solution was centrifuged and the corneal stromal cells (CSCs) were resuspended in DMEM/F12 medium containing 5 % FBS and 1 % penicillin-streptomycin (PS, Gibco). CSCs were cultured at 37 °C in 5 % CO2 and passaged at a 1:3 ratio every 3 days using 0.25 % trypsin.

Human corneal epithelial cells (HCEpC) and corneal endothelial cells (HCEnC) were derived from immortalized cell lines. The HCEpC cell line, HCE-T, was a gift from Dr. Shengguo Li, and the HCEnC cell line, HCEC-B4G12, was purchased from Shanghai Hongshun Biotechnology Co., Ltd. Both cell types were cultured in DMEM/F12 medium containing 10 % FBS and 1 % PS at 37 °C in 5 % CO_2_ and were passaged at a 1:3 ratio every 3 days using 0.25 % trypsin.

**Fig. 1:**
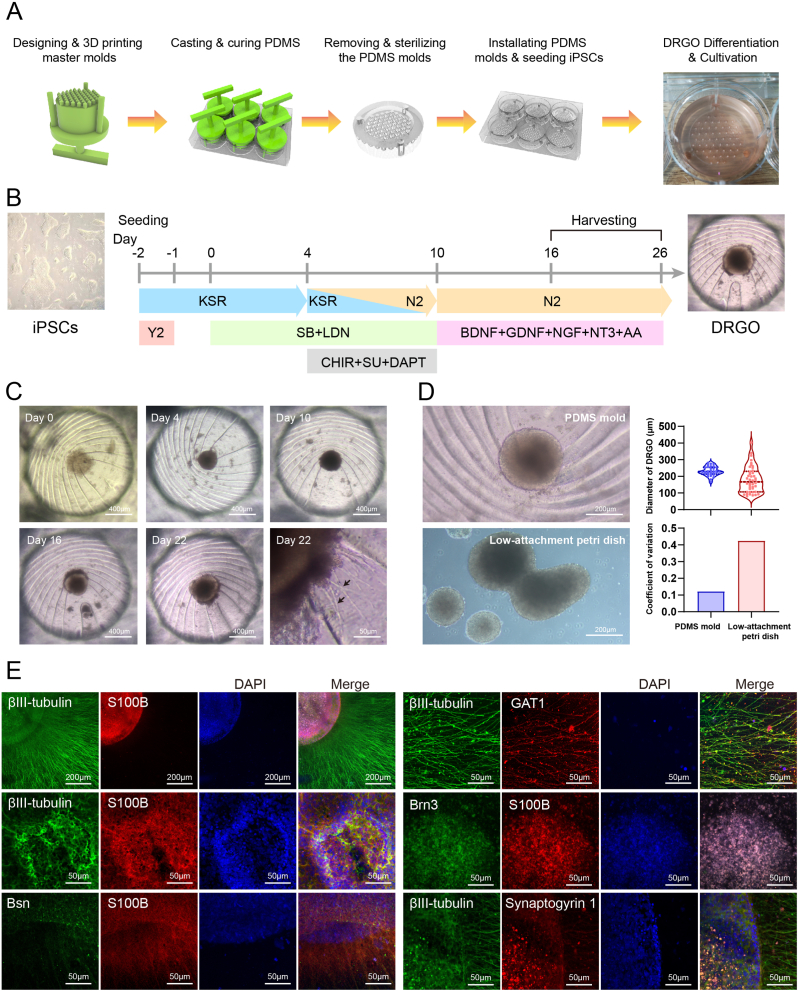
Differentiation and Characterization of Dorsal Root Ganglion Organoids (DRGO). (A) Schematic of the process for designing and 3D printing master molds, casting and curing PDMS, removing and sterilizing PDMS molds, and installing PDMS molds to seed iPSCs for DRGO differentiation. (B) Timeline and protocol for differentiating iPSCs into DRGOs. (C) Morphological changes in DRGOs at different time points during differentiation (Days 0, 4, 10, 16, and 22). By Day 22, nerve fiber extensions are visible (black arrows). (D) Comparison of DRGOs cultured in PDMS molds and low-attachment Petri dishes. DRGOs in PDMS molds exhibit more uniform size and a lower coefficient of variation than those in low-attachment Petri dishes. (E) Immunofluorescence staining of DRGOs. βIII-tubulin (green) and S100B (red) identify neurons and glial cells, respectively. Additional markers GAT1, Brn3, Bsn, and Synaptogyrin 1 characterize various neuronal subtypes and synaptic components. Nuclei are counterstained with DAPI (blue). (For interpretation of the references to colour in this figure legend, the reader is referred to the Web version of this article.)

Urine-derived hiPS cell lines were purchased from Cellapy Biotechnology, Beijing, China. hiPSCs were maintained on 6-well plates (Corning, USA) using mTeSR Plus medium (STEMCELL Technologies) and coated with Laminin-521 (LN-521, BioLamina). Cells were cultured at 37 °C and 5 % CO_2_ and passaged at a 1:8 ratio every 3–4 days using 0.5 mM EDTA (Cellapy Biotechnology). The differentiation process for dorsal root ganglion organoids (DRGOs) is illustrated in Fig. 1A and B. In brief, we utilized a previously described innovative culture method, employing 3D-printed photopolymer positive molds to create PDMS negative molds for organoid culture, as detailed in our prior study [32]. The specific parameters of the molds are shown in Fig. S1. Each PDMS mold contains 61 v-shaped bottom wells. After autoclaving the PDMS molds, they were placed into 6-well plates, and 1 ml of a suspension containing 1 × 10^5^ iPSC cells was added to each mold. The plates were centrifuged at 100×*g* for 3 min. The differentiation protocol followed the method described by Mazzara et al. [33] with some modifications. On Day −2, 3 ml of KSR medium containing 100 μM Y27632 was added, which included DMEM-F12, 15 % KSR, 1 % PS, non-essential amino acids (NEAA, Thermo 11140035), 100 μM β-mercaptoethanol (Thermo 31350010), and 2 mM L-glutamine. On Day −1, the medium was replaced with KSR medium without Y27632. On Day 0, the KSR medium was supplemented with 10 μM SB431542 (MCE, HY-10431) and 100 μM LDN193189 (MCE, HY-12071). The medium was changed every 48 h. From Day 4 to Day 9, in addition to SB and LDN, 3 μM CHIR99021 (MCE, HY-10182), 3 μM SU5402 (MCE, HY-10407), and 10 μM DAPT (MCE, HY-13027) were added. During this period, the culture medium was gradually switched from KSR to N2 medium (Neurobasal medium, Thermo 21103049), 1 % N2 supplement (Thermo 17502-048), 1 % PS, 1 % NEAA, and 2 mM L-glutamine. On Day 10, the medium was replaced with N2+ medium, which included 100 % N2 medium supplemented with recombinant human brain-derived neurotrophic factor (BDNF, Peprotech 450-02) at 10 ng/ml, glial cell-derived neurotrophic factor (GDNF, Peprotech 450-10) at 10 ng/ml, nerve growth factor (NGF, Merck N6009) at 10 ng/ml, neurotrophin-3 (NT-3, Peprotech 450-03) at 10 ng/ml, and 200 μM ascorbic acid (AA, Merck 49752). The medium was changed every 2.5 days. Differentiated DRGOs were harvested between Days 16 and 26 for subsequent co-culture model construction.

### 2.3 Construction of a biomimetic full-thickness corneal model *in vitro*

**Fig. 2:**
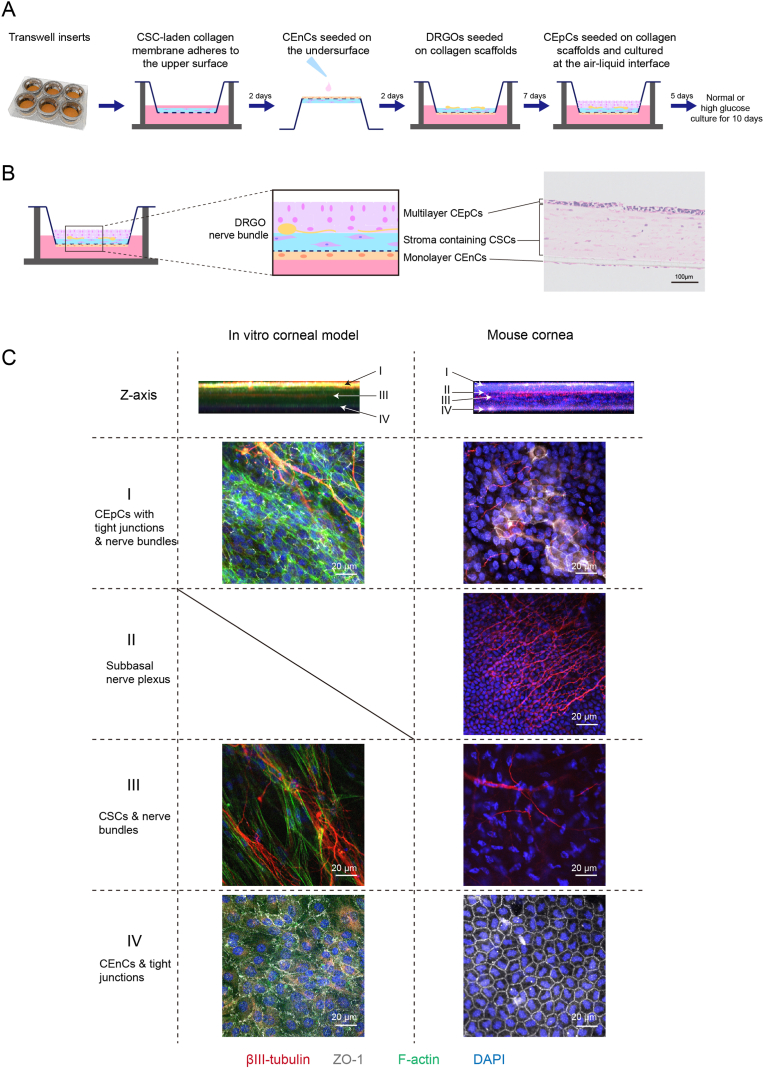
Construction and Characterization of the Full-Thickness Corneal Model. (A) Schematic of the construction process for the full-thickness corneal model. The process involves adhering a CSC-laden collagen membrane to the upper surface, seeding CEnCs on the undersurface, seeding DRGOs on collagen scaffolds, and finally seeding CEpCs on top. The model is cultured at the air-liquid interface and exposed to either normal or high glucose conditions for 10 days. (B) Cross-sectional schematic and H&E staining image showing the structure of the full-thickness corneal model, including a monolayer of CEnCs, stroma containing CSCs, multilayer CEpCs, and integrated DRGO nerve bundles. The immunofluorescence images show the Z-axis structure and cellular characteristics of each layer in the full-thickness *in vitro* corneal model and the mouse cornea (C). βIII-tubulin (red) marks nerve bundles, F-actin (green) shows the cytoskeleton, and ZO-1 (grey) highlights tight junctions. DAPI (blue) marks the nuclei. The scale bars represent 20 μm. (For interpretation of the references to colour in this figure legend, the reader is referred to the Web version of this article.)

The construction process of the model is illustrated in Fig. 2A. Collagen membranes containing corneal stromal cells (CSCs) were prepared using our previously established methods [22,34]. Briefly, 4 × 10^6^ third-passage CSCs were collected and mixed with 9 mL of 0.5 % pre-cooled type I collagen solution (extracted from bovine tendon, Guangzhou Trauer Biotechnology, China) and 1 mL of 10 × DMEM (Hyclone Laboratories, Logan, UT, USA). The mixture was thoroughly combined and centrifuged to remove air bubbles. The collagen solution containing CSCs was then poured into a 12-well plate, with 1 mL per well. The plate was incubated at 37 °C for 30 min to allow the collagen solution to solidify into a collagen gel. A 500-mesh nylon net was placed on both sides of the collagen gel, and absorbent filter paper was positioned around the perimeter of the nylon net. A glass plate was placed on top of the filter paper, and a 100 g weight was applied to compress the collagen gel into a collagen membrane, which took approximately 10–20 min. The collagen membrane was then placed on the upper surface of a transwell insert in a six-well plate, ensuring tight adherence. Next, 1 mL of DMEM/F12 medium containing 10 % FBS was added to the transwell insert, and the CSCs were cultured at 37 °C and 5 % CO_2_ for 2 days to ensure cell survival. After 2 days, the transwell insert was inverted, exposing the bottom surface. The bottom surface was coated with PBS solution containing laminin 521 (Thermo, A29249) and collagen IV (Merck, C5533) at 37 °C for 1 h, using concentrations of 0.75 μg/cm^2^ laminin-521 and 5 μg/cm^2^ collagen IV. Then, 2 × 10^6^ CEnCs/mL were seeded onto the bottom surface and cultured at 37 °C and 5 % CO2 for 1 h to allow cell adhesion. The transwell insert was then placed in a six-well plate and cultured in DMEM/F12 medium containing 10 % FBS for an additional 2 days to allow the endothelial cells to form a monolayer. Next, 15 DRGOs were extracted from the PDMS molds and placed onto the collagen membrane containing CSCs, ensuring even distribution. N2+ medium was added to the lower chamber of the transwell, while the upper chamber remained empty. The culture was maintained for 7 days to allow the nerve bundles to grow. After 7 days, 3 × 10^6^ CEpCs/mL were collected and 1 mL was seeded onto the upper surface of the transwell insert. After 1 day, once the cells adhered, the medium in the upper chamber was aspirated, and 2 mL of DMEM/F12 medium containing 10 % FBS was added to the lower chamber. The model was cultured at an air-liquid interface for 5 days to form a multilayered epithelial layer. The model was then cultured in either normal control (NC), high glucose (HG), or high glucose plus Lycium barbarum glycopeptide (HG + LBGP) medium for an additional 10 days to construct the experimental models. The working concentration of HG was 65 mM. LBGP was provided by Tianren Goji Biotechnology Co., Ltd, Ningxia, China.

### 2.4 CCK8 assay

To evaluate the activity and toxicity of LBGP in CEpC cells, a CCK8 assay was performed. CEpC cells were seeded at a density of 5 × 10^3^ cells/well in a 96-well plate and cultured for 24 h to allow cell adhesion. Subsequently, various concentrations of LBGP (0–1000 μg/mL) were added to each well and incubated at 37 °C with 5 % CO₂ for 24 h. Then, 10 μL of CCK8 reagent was added to each well, and the plate was incubated for an additional 2 h. Finally, the absorbance of each well was measured at 450 nm using a microplate reader. Each experimental group included three parallel replicates to ensure the reliability of the results. Cell viability was calculated based on the absorbance values to assess the activity and toxicity of LBGP.

### 2.5 Scratch assay

To examine the effect of LBGP on the activity and migration of CEpC cells, a scratch assay was conducted. CEpCs were seeded at a density of 2 × 10^5^ cells/well in a 6-well plate and cultured until confluent. A sterile 200 μL pipette tip was used to make a uniform scratch across the center of each well. Cells were gently washed with PBS to remove suspended cells. Then, a serum-free medium containing various concentrations of LBGP was added to the experimental groups, while the control group received a serum-free medium only. HG (65 mM) was used as a damage model. Cells were cultured at 37 °C with 5 % CO₂. Images of the scratch area were captured at 0 h and 24 h using a microscope. The scratch area was measured to evaluate the effect of LBGP on CEpC cell migration by comparing the changes in the scratch area between 0 h and 24 h. Each experimental group included three parallel replicates to ensure the reliability of the results.

### 2.6 TEM and SEM

The internal and surface microstructures of the samples were observed using transmission electron microscopy (TEM) and scanning electron microscopy (SEM). Samples were prepared as previously described. Briefly, samples (approximately 2 × 2 mm in size) were fixed with 2.5 % glutaraldehyde for 2 h, followed by fixation with 2 % osmium tetroxide for 1 h, and then dehydrated through a graded ethanol series. Ultrathin sections (70–90 nm thick) were cut using an ultramicrotome, placed on TEM grids, and stained at room temperature with 2 % uranyl acetate, 1 % phosphotungstic acid, and Reynolds' lead citrate. Images were captured using a transmission electron microscope (Hitachi H-7600, Hitachi, Japan). For SEM, samples were fixed with 2.5 % glutaraldehyde for 2 h, washed three times with PBS, dehydrated through a graded ethanol series for 10 min each, coated with gold-palladium alloy, and observed using a scanning electron microscope (JSM-T300-SEM, JEOL Technics Co. Ltd., Tokyo, Japan).

### 2.7 Immunofluorescence and H&E staining

Samples or frozen sections were fixed with 4 % paraformaldehyde at room temperature for 15 min. After rinsing three times with PBS, the samples were incubated in a blocking and permeabilization solution containing 3 % BSA and 1 % Triton-X 100 at room temperature for 1 h. Primary antibodies (mouse monoclonal antibody βIII-tubulin, 1:1000, Abcam; rabbit polyclonal antibody S100B, 1:500, Bioss; rabbit polyclonal antibody Synaptogyrin 1, 1:500, Synaptic Systems; rabbit polyclonal antibody GABA transporter 1 (GAT 1), 1:500, Synaptic Systems; mouse polyclonal antibody Brn3, 1:500, Santa Cruz Biotechnology; mouse polyclonal antibody bassoon (Bsn), 1:200, Santa Cruz Biotechnology; rabbit polyclonal antibody ZO-1, 1:500, Abcam; mouse polyclonal antibody RANTES, 1:200, Santa Cruz Biotechnology; rabbit polyclonal antibody Nrf2, 1:200, Abcam; mouse polyclonal antibody ICAM-1, 1:200, Santa Cruz Biotechnology; rabbit polyclonal antibody HMOX1, 1:500, Proteintech; rabbit polyclonal antibody iNOS, 1:500, Proteintech; rabbit polyclonal antibody α-SMA, 1:500, Proteintech) were added to the solution, and the samples were incubated overnight at 4 °C. After washing with PBS, the samples were incubated in a solution containing 1 % BSA and 1:1000 secondary antibodies (goat anti-rabbit/mouse IgG Alexa Fluor 488/594, Invitrogen, Carlsbad, CA, USA) at room temperature for 1.5 h. The nuclei were then stained with DAPI. After washing with PBS, images of the samples were captured using a laser scanning confocal microscope (LSM 800, Zeiss, Germany).

The tissue samples were fixed with 4 % paraformaldehyde at room temperature for 15–30 min, then rinsed three times with PBS for 5 min each. The fixed tissue samples were dehydrated in PBS containing 30 % sucrose until the samples sank. The samples were then embedded in OCT and rapidly frozen. Using a cryostat, the samples were sectioned into 5–10 μm thick slices and placed on pre-cooled slides. After air-drying, the sections were sequentially stained in the following solutions: 75 % ethanol for 2 min, hematoxylin solution for 5–10 min, rinsed with tap water until clear, 1 % hydrochloric acid ethanol for a few seconds, rinsed with tap water until clear, ammonia water or sodium bicarbonate solution for 1–2 min, rinsed with tap water for 5 min, eosin solution for 30 s to 1 min, and quickly rinsed with tap water. The sections were then dehydrated and mounted as follows: 75 % ethanol for 2 min, 95 % ethanol for 2 min, 100 % ethanol twice for 2 min each, and xylene twice for 2 min each. Finally, the coverslips were mounted with neutral gum to avoid air bubbles. The prepared samples were then observed and imaged using a microscope.

### 2.8 Cytokine array

The GSH-CAA-2000 cytokine microarray (RayBiotech, USA) was used for the semi-quantitative detection of cytokines in the culture medium. Slides were equilibrated at room temperature for 20–30 min and dried in a vacuum desiccator for 1–2 h. After blocking with 100 μL of sample dilution, 100 μL of standards and samples were added and incubated overnight at 4 °C. Slides were washed with 1 × Wash Buffer I and II, and detection antibodies were added and incubated for 2 h. After another wash, Cy3-streptavidin was added, and slides were incubated in the dark for 1 h. Finally, signals were scanned at 532 nm using a laser scanner, and data were analyzed with GSH-CAA-2000 software. Cytokines with average signal values greater than 1.2-fold and p-values less than 0.05 were considered differentially expressed. Enrichment analysis was performed using Metascape (www.metascape.org) [35].

### 2.9 RNA sequencing (RNA-Seq)

Four types of cells were extracted from the samples. DRGOs were separated with forceps, while CEpCs and CEnCs were isolated by adding 0.25 % trypsin to the upper and lower chambers of the transwell. CSCs were collected by digesting the remaining collagen membrane with 0.5 % Type I collagenase (Gibco, 17100017). The collected cells were then treated with TRIzol (Thermo Fisher Scientific, USA) to extract RNA. Samples were divided into NC and HG groups, with three biological replicates per group. cDNA libraries were sequenced on the Illumina platform by Genedenovo Biotechnology Co., Ltd (Guangzhou, China). After library sequencing and alignment, gene counts were calculated. Principal component analysis (PCA) was performed, and counts per million (CPM) were calculated to standardize gene expression levels. Data normalization and differential expression gene (DEG) screening were performed using the R package edgeR (3.26.8) (www.r-project.org), with thresholds set at fold change >1.5 or < −1.5 and false discovery rate (FDR) < 0.05. Gene ontology (GO) enrichment analysis and Kyoto Encyclopedia of Genes and Genomes (KEGG) analysis were conducted using Metascape (www.metascape.org). Venn plots, protein-protein interaction (PPI) networks and hub gene analysis were also performed.

### 2.10 qPCR

The extracted RNA was reverse transcribed into cDNA using a reverse transcriptase (Vazyme, China). The qPCR reaction system, totaling 10 μL, contained 5 μL 2X SYBR (Vazyme, China), 250 nM forward and reverse primers, and 10 ng cDNA template. Primers were designed using Primer-BLAST (NCBI) and are listed in Table S1. qPCR amplification was performed using the LightCycler96 (Roche, Switzerland). Each amplification result was normalized to GAPDH mRNA transcripts. Gene transcript expression changes in each sample were calculated using the 2^−ΔΔCt method. Results from three independent experiments were statistically analyzed, with at least three parallel replicates per experiment.

### 2.11 TEER measurement

To assess the barrier function of the model, the transepithelial electrical resistance (TEER) of the samples was measured using a Millicell-ERS-2 volt-ohm meter (Millipore, USA) according to the manufacturer's instructions. The samples were placed in a six-well plate transwell with equal amounts of culture medium added to both the inner and outer chambers. Electrodes were inserted into the inner and outer chambers to detect resistance changes. TEER values were calculated using the formula: TEER (Ω·cm^2^) = R × A, where R represents the measured resistance (Ω), and A represents the membrane area of each insert (cm^2^). The experiment was performed in at least three parallel replicates.

### 2.12 Western blot analysis

Total protein was extracted from cells or tissues using RIPA lysis buffer, and protein concentration was determined by the BSA method. Equal amounts of protein samples were separated by SDS-PAGE and then transferred to PVDF membranes. The membranes were blocked with TBST buffer containing 5 % non-fat milk for 1 h to prevent nonspecific binding. Membranes were then incubated overnight at 4 °C with primary antibodies (rabbit polyclonal anti-HMOX1, 1:500, Proteintech; mouse monoclonal anti-ICAM-1, 1:500, Santa Cruz Biotechnology; mouse monoclonal anti-GAPDH, 1:500, Proteintech). The next day, membranes were washed three times with TBST for 10 min each, followed by incubation with appropriate secondary antibodies at room temperature for 1 h. Membranes were washed again three times with TBST for 10 min each. Finally, bands were visualized using chemiluminescent substrates and recorded with an imaging system. The experiment was performed in at least three parallel replicates.

### 2.13 TUNEL assay

Apoptosis was analyzed using a TUNEL assay kit (Beyotime Biotechnology, Shanghai, China). Samples were fixed with 4 % paraformaldehyde at room temperature for 15 min, followed by blocking and permeabilization with a solution of 3 % BSA and 1 % Triton-X 100 at room temperature for 1 h. Samples were then incubated with TUNEL detection solution at 37 °C for 1 h. Nuclei were stained with DAPI. After washing with PBS, images of the samples were captured using a fluorescence microscope. The experiment was performed in at least three parallel replicates.

### 2.14 ROS measurement

To measure intracellular reactive oxygen species (ROS), DCFH-DA dye was used. Cells were cultured to the logarithmic growth phase and treated accordingly. DCFH-DA dye (10 μM, Beyotime Biotechnology, Shanghai, China) was diluted in serum-free medium. Treated cells were washed twice with PBS, then incubated with the diluted DCFH-DA working solution for 30 min at 37 °C in the dark. After incubation, cells were washed three times with PBS to remove any unbound dye. Finally, the fluorescence intensity of intracellular ROS was detected using a fluorescence microscope. The experiment was performed in at least three parallel replicates to ensure the reliability of the results.

### 2.15 ELISA

The concentrations of IL-6 and RANTES in the sample culture medium were determined by enzyme-linked immunosorbent assay (ELISA) using human IL-6 and RANTES ELISA kits (Absin, China). The assay was performed according to the manufacturer's instructions. Absorbance was measured using a microplate reader (BioTek, VT, USA). The experiment was performed in at least three parallel replicates.

### 2.16 Animal experiments

Eight-week-old male db/db mice and C57BL/6 mice were obtained from SJA Laboratory Animal Co., Hunan, China, and housed under controlled lighting conditions (12-h light/dark cycle) with free access to food and water. Mice with blood glucose levels higher than 16.7 mmol/L were considered diabetic mellitus. The 24 mice were randomly divided into three groups (n = 8 per group): control, db/db, and db/db + LBGP. LBGP was dissolved in 0.01 M PBS at room temperature. The db/db + LBGP group received LBGP via oral gavage at a dose of 50 mg/kg daily. The control and db/db groups received daily gavage with 0.01 M PBS. After three months of intervention, the mice were used for experiments. Following euthanasia, the corneas were carefully excised along the limbus. The samples were incubated at room temperature for 1 h in a blocking and permeabilization solution containing 3 % BSA and 1 % Triton X-100. Subsequently, the samples were incubated overnight at 4 °C in a 1 % BSA solution containing 1:100 anti-βIII tubulin Alexa Fluor® 488 conjugate (Millipore, USA) and thoroughly washed with PBS. Four radial cuts were made in each cornea for imaging, and the tissue was flat-mounted on slides with the epithelium facing up. Images were captured using a laser scanning confocal microscope (LSM 800, Zeiss, Jena, Germany). The remaining corneas were used for cryosectioning. Fixed corneal tissues were dehydrated in a PBS solution containing 30 % sucrose until they sank. The corneal tissues were then embedded in an OCT compound and rapidly frozen. Sections were cut into 5–10 μm thick slices using a cryostat and placed on pre-cooled slides. After air drying, the sections were incubated at room temperature for 1 h in a blocking and permeabilization solution containing 3 % BSA and 0.3 % Triton X-100. The sections were then incubated overnight at 4 °C with primary antibodies. The next day, the sections were washed three times with PBS for 5 min each, incubated with fluorescently labeled secondary antibodies at room temperature for 1 h in the dark, and washed three times with PBS for 5 min each. The nuclei were stained with DAPI. Finally, images were captured using a fluorescence microscope.

### 2.17 Statistical analysis

Data were expressed as mean ± standard deviation (SD). Comparisons between groups were performed using unpaired two-tailed t-tests with GraphPad 8.0 (GraphPad Software, Inc., La Jolla, CA, USA). A P-value <0.05 was considered statistically significant. Fluorescence intensity, cell counts, nerve length and junction counts, area calculations, length measurements, and grayscale analysis were performed using Fiji/ImageJ (National Institutes of Health, MD, USA).

## 3 Results

### 3.1 Efficient generation of uniform DRGOs

In previous research, we designed a novel organoid culture mold [32]. The master mold was fabricated using a light-sensitive resin 3D printing method, and the culture mold was produced using PDMS casting. A physical representation of the mold is shown in Fig. 1A, with detailed parameters provided in Fig. S1. The mold consists of 61 small wells with V-shaped bottoms, connected by channels to facilitate rapid liquid infiltration. After autoclaving, the mold was placed into a 6-well plate, and 1 × 10^5^ iPSCs were seeded to initiate the differentiation of DRGOs. By day 16 of differentiation, DRGOs had formed, as depicted in Fig. 1B. Microscopic images show a clear neuronal region surrounding the DRGOs by day 16, and by day 22, nerve bundles were visible (Fig. 1C). The diameter of the organoids was measured at day 22, revealing that DRGOs constructed using the PDMS mold had a diameter of 230.8 ± 27.49 μm, while those formed using low-attachment petri dishes had a diameter of 180.1 ± 76.96 μm. Compared to traditional culture methods, organoids produced using this novel mold exhibited a lower coefficient of variation (0.119 vs. 0.427), indicating more consistent organoid sizes. Additionally, the cell spheroids did not fuse together (Fig. 1D). After day 22, DRGOs were cultured in collagen gel for 7 days. Immunofluorescence identification revealed that DRGOs expressed neuronal markers such as βIII-tubulin, glial cell marker S100B, GABA transporter protein GAT1, presynaptic membrane marker Bassoon (Bsn), sensory neuron marker Brn3, and synaptic vesicle-associated protein Synaptogyrin 1 (Fig. 1E). These results indicate that differentiated DRGOs possess the structure and function of sensory neurons. This method enables the efficient and large-scale differentiation of homogeneous DRGOs, providing sensory neurons for the construction of *in vitro* models.

### 3.2 Construction of a full-thickness *in vitro* corneal model

This *in vitro* corneal model was constructed using transwell inserts as the core component. The detailed construction method is described in the Methods section. The construction process is illustrated in Fig. 2A. After 16 days of cultivation, the *in vitro* corneal model was completed. Hematoxylin and eosin (H&E) staining revealed a multilayered corneal epithelium, a stromal layer containing corneal stromal cells, and a monolayer of corneal endothelial cells (Fig. 2B). Immunofluorescence staining demonstrated that individual DRGOs formed long nerve bundles (βIII-tubulin, red), which integrated with the corneal epithelial cells and anterior stroma (cytoskeleton F-actin, green) (Fig. 2C I, II). The corneal epithelium displayed tight junctions (ZO-1, grey), and the monolayer corneal endothelial cells also exhibited tight junction structures (Fig. 2C I, IV). Comparisons with mouse corneas revealed a high degree of similarity between the *in vitro* model and the natural cornea. The *in vitro* model successfully recapitulated the integration of corneal nerves with epithelial cells and the formation of tight junctions (Fig. 2C I), as well as the innervation of the anterior stroma by corneal nerves (Fig. 2C III). The endothelial layer in the *in vitro* model formed a tight monolayer consistent with the mouse cornea (Fig. 2C IV). However, the subbasal nerve plexus structure, prominent in mouse corneas, was absent in the *in vitro* model (Fig. 2C II). This full-thickness *in vitro* corneal model exhibits structural characteristics similar to physiological cornea, providing a valuable tool for *in vitro* research on corneal diseases.

### 3.3 High-glucose environment induces cell damage in the full-thickness biomimetic corneal model

**Fig. 3:**
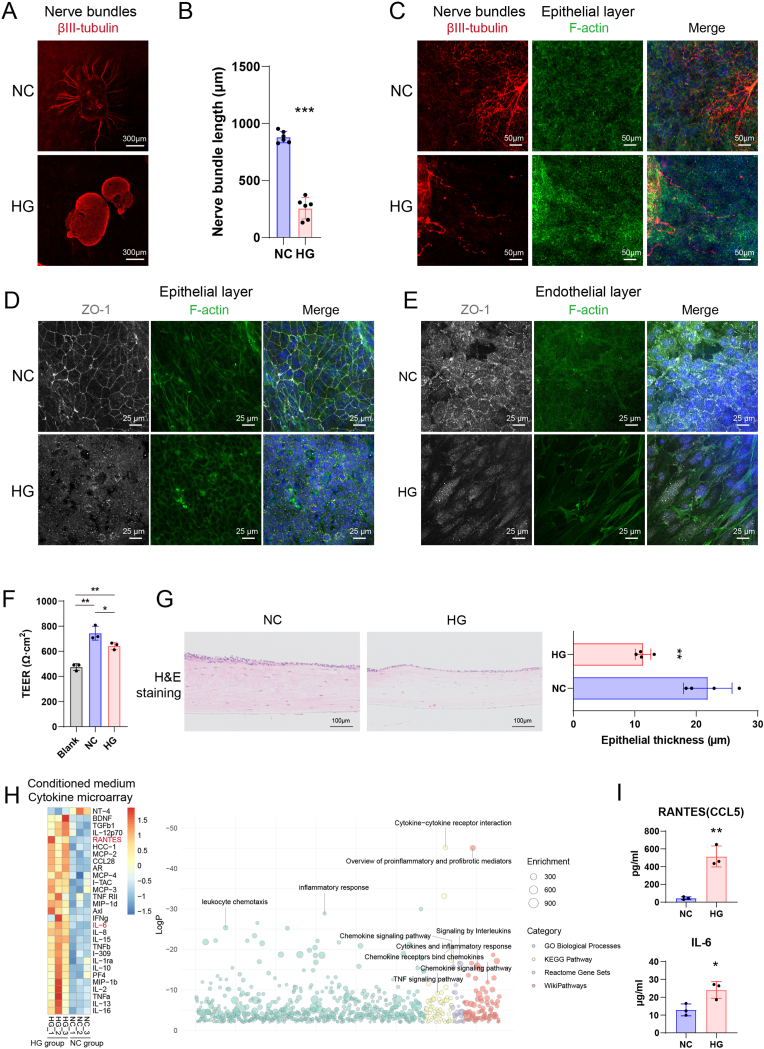
Effects of HG Environment on the Full-Thickness Corneal Model. (A) Immunofluorescence staining of βIII-tubulin showing nerve bundles in normal control (NC) and high glucose (HG) groups. (B) Quantification of nerve bundle length in NC and HG groups. (C) Immunofluorescence staining of βIII-tubulin (red) and F-actin (green) in the corneal epithelial layer. (D) Immunofluorescence staining of ZO-1 (white) and F-actin (green) in the corneal epithelial layer. (E) Immunofluorescence staining of ZO-1 (white) and F-actin (green) in the corneal endothelial layer. (F) Comparison of transepithelial electrical resistance (TEER) measurements among NC, HG, and blank groups. (G) Hematoxylin and eosin (H&E) staining of corneal epithelium, quantifying epithelial thickness in NC and HG groups. (H) Cytokine microarray analysis of conditioned medium from NC and HG groups, including heatmap and enrichment analysis. (I) ELISA quantification of RANTES (CCL5) and IL-6 levels in conditioned medium from NC and HG groups. (∗∗∗, p < 0.001; ∗∗, p < 0.01; ∗, p < 0.05). (For interpretation of the references to colour in this figure legend, the reader is referred to the Web version of this article.)

The *in vitro* corneal models were induced using high-glucose (HG) or normal culture (NC) conditions. After 14 days of cultivation, relevant indicators were assessed. βIII-tubulin staining was used to visualize corneal nerve bundles. The results showed that under NC conditions, nerve bundles grew normally with an extension length of 880.8 ± 46.38 μm. In contrast, HG induction caused the disintegration of corneal nerve bundles (Fig. 3A), significantly reducing their length to 255.7 ± 85.53 μm (Fig. 3B). Local images revealed that in the NC group, nerves innervated corneal epithelial cells, whereas in the HG group, disintegrated corneal nerves disappeared among the epithelial cells (Fig. 3C). Examination of the epithelial layer cells showed that the tight junction marker ZO-1 was clearly visible between cells in the NC group but completely disappeared under HG conditions (Fig. 3D). Similar results were observed in the endothelial layer: ZO-1 was present between endothelial cells in the NC group but not apparent in the HG group, where endothelial cells also adopted a fibrotic shape (Fig. 3E). Barrier function assessment showed that the TEER values for blank transwells (Blank), NC, and HG were 474.8 ± 22.01 Ω cm^2^, 744.1 ± 45.29 Ω cm^2^, and 643.7 ± 23.06 Ω cm^2^, respectively, indicating that HG compromised the corneal barrier function (Fig. 3F). Furthermore, the thickness of the corneal epithelium changed under HG conditions. H&E staining showed that the epithelial thickness in the NC group was 21.88 ± 3.41 μm, while in the HG group, it dramatically decreased to 11.35 ± 1.10 μm (Fig. 3G).

Conditioned medium was collected from NC and HG groups for cytokine detection using a cytokine microarray. These two groups showed obvious intergroup differences (Fig. S2A). Out of 120 cytokines tested, 29 showed significant differences. Among these, 28 cytokines were upregulated in the HG group, with the neurotrophic factor NT-4 downregulated (Fig. S2B). Enrichment analysis of the upregulated factors highlighted their involvement in leukocyte chemotaxis, inflammatory response, and TNF signaling pathway, among others (Fig. 3H). ELISA validation confirmed that RANTES (CCL5) and IL-6 were markedly upregulated in the HG group. The concentrations of RANTES in the NC and HG groups were 44.76 ± 12.99 pg/ml and 515.1 ± 96.16 pg/ml, respectively. IL-6 concentrations in the NC and HG groups were 18.02 ± 3.83 μg/ml and 9.67 ± 2.74 μg/ml, respectively (Fig. 3I).

**Fig. 4:**
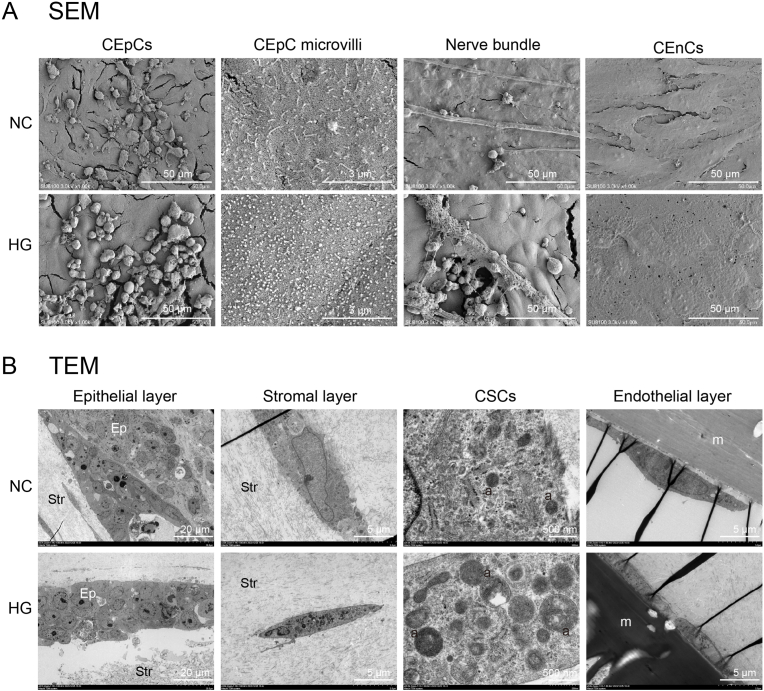
Ultrastructural Analysis of the Full-Thickness Corneal Model in NC and HG Groups. (A) Scanning electron microscopy (SEM) images of corneal epithelial cells (CEpCs), CEpC microvilli, nerve bundles, and corneal endothelial cells (CEnCs) under normal control (NC) and high glucose (HG) conditions. (B) Transmission electron microscopy (TEM) images of the corneal epithelial layer, stromal layer, corneal stromal cells (CSCs), and endothelial layer in NC and HG groups.

The microscopic structure of the samples was examined using scanning electron microscopy (SEM) and transmission electron microscopy (TEM). SEM images revealed that, compared to the NC group, the HG group exhibited an increased number of dead corneal epithelial cells, shortened corneal epithelial microvilli, and abnormal extracellular matrix (ECM) attachments on nerve bundles. Additionally, the number of corneal endothelial cells decreased in the HG group, and they failed to cover the pores on the transwell membrane, disrupting the monolayer structure of the cells (Fig. 4A). TEM results showed that, compared to the NC group, the thickness of the corneal epithelial layer was reduced in the HG group. An increased number of autophagosomes and larger autophagosome sizes were observed in corneal stromal cells in the HG group (Fig. 4B). These findings indicate that HG enhances the immune response and causes cellular damage in the full-thickness biomimetic corneal model.

### 3.4 Transcriptomic changes between NC and HG conditions

**Fig. 5:**
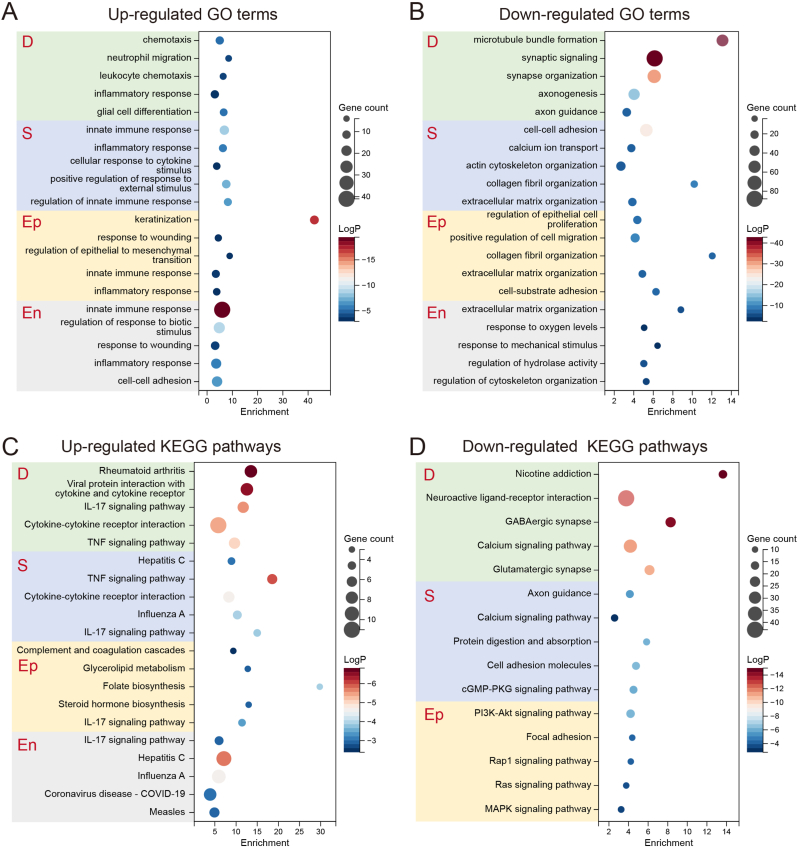
GO and KEGG Pathway Enrichment Analysis. (A) Up-regulated GO terms under high glucose (HG) conditions, categorized by cell type: dorsal root ganglion organoids (D), stromal cells (S), epithelial cells (Ep), and endothelial cells (En). (B) Down-regulated GO terms under HG conditions, categorized by cell type. (C) Up-regulated KEGG pathways under HG conditions, categorized by cell type. (D) Down-regulated KEGG pathways under HG conditions, categorized by cell type.

To investigate the transcriptomic changes between normal culture (NC) and high-glucose (HG) conditions, we isolated four types of corneal cells from the *in vitro* corneal model: corneal epithelial cells (CEpCs, Ep), corneal stromal cells (CSCs, S), corneal endothelial cells (CEnCs, En), and dorsal root ganglion organoids (DRGOs, D). RNA was extracted from these cells for sequencing. Principal component analysis (PCA) indicated distinct intergroup differences (Fig. S3A). Compared to NC, HG resulted in the downregulation of 436 genes and upregulation of 71 genes in DRGOs (D), downregulation of 410 genes and upregulation of 44 genes in S, downregulation of 176 genes and upregulation of 51 genes in Ep, and downregulation of 37 genes and upregulation of 170 genes in En (Fig. S3B). Gene Ontology (GO) enrichment and KEGG pathway analyses were performed on these differentially expressed genes (DEGs). Upregulated GO terms in D included leukocyte chemotaxis, inflammatory response, and glial cell differentiation. In S, upregulated GO terms included innate immune response, inflammatory response, and cellular response to cytokine. In Ep, upregulated GO terms included keratinization, regulation of epithelial to mesenchymal transition, and inflammatory response. In En, upregulated GO terms included response to wounding, inflammatory response, and cell-cell adhesion (Fig. 5A). Downregulated GO terms in D included synaptic signaling, synapse organization, and axonogenesis. In S, downregulated GO terms included collagen fibril organization, extracellular matrix organization, and cell-cell adhesion. In Ep, downregulated GO terms included regulation of epithelial cell proliferation, collagen fibril organization, and cell-substrate adhesion. In En, downregulated GO terms included extracellular matrix organization, response to oxygen levels, and regulation of hydrolase activity (Fig. 5B). KEGG pathway enrichment analysis showed that upregulated pathways in D included the IL-17 signaling pathway and TNF signaling pathway. In S, upregulated pathways included Hepatitis C, TNF signaling pathway, and IL-17 signaling pathway. In Ep, upregulated pathways included complement and coagulation cascades and IL-17 signaling pathway. In En, upregulated pathways included the IL-17 signaling pathway and Hepatitis C (Fig. 5C). Downregulated pathways in D included neuroactive ligand-receptor interaction, GABAergic synapse, and calcium signaling pathway. In S, downregulated pathways included the calcium signaling pathway, protein digestion and absorption, and cell adhesion molecules. In Ep, downregulated pathways included the PI3K-Akt signaling pathway, focal adhesion, and MAPK signaling pathway (Fig. 5D). These analyses indicate that HG induces an increase in inflammation and immune response in corneal cells, impairs axon and synapse formation, collagen synthesis in stromal and epithelial cells, and the antioxidative stress response in epithelial and endothelial cells.

**Fig. 6:**
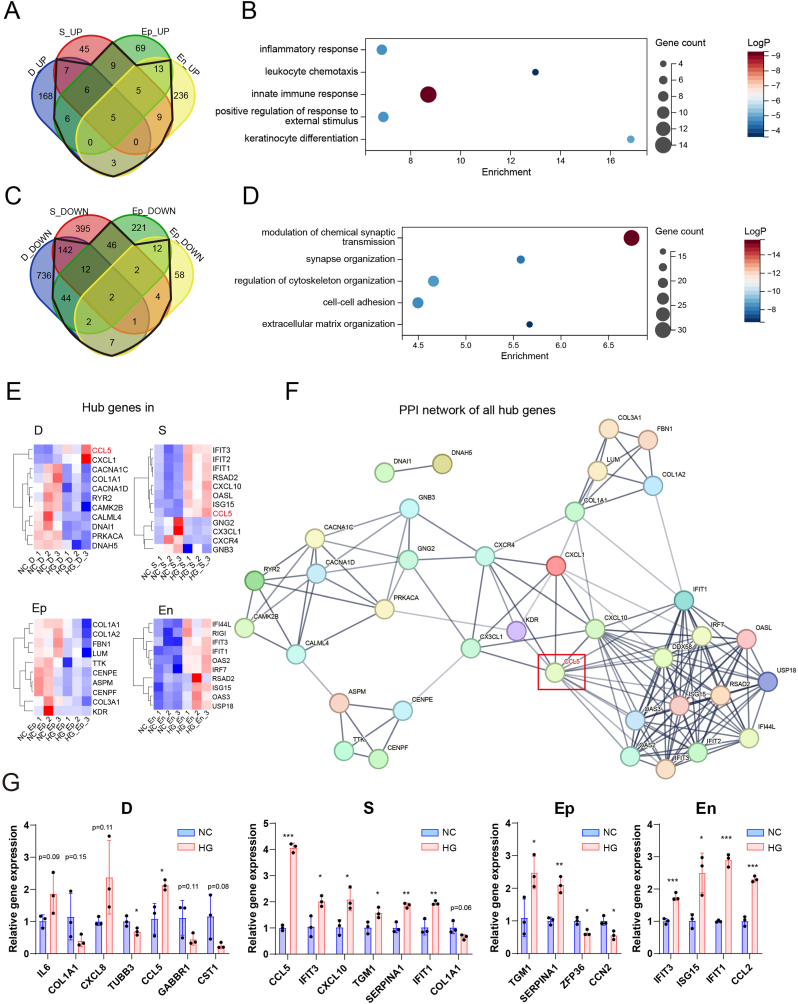
Co-Expression of Differentially Expressed Genes and Protein-Protein Interaction (PPI) Analysis. (A) Venn diagram of up-regulated genes in dorsal root ganglion organoids (D), stromal cells (S), epithelial cells (Ep), and endothelial cells (En) under HG conditions. (B) Enrichment analysis of up-regulated GO terms shared by multiple cell types under HG conditions. (C) Venn diagram of down-regulated genes in D, S, Ep, and En cells under HG conditions. (D) Enrichment analysis of down-regulated GO terms shared by multiple cell types under HG conditions. (E) Heatmap showing hub genes in D, S, Ep, and En cells. (F) Protein-protein interaction (PPI) network of all hub genes. (G) qPCR validation of relative gene expression levels of selected genes in D, S, Ep, and En cells, comparing NC and HG groups. Data are presented as mean ± standard deviation.

The gene expression profiles of four corneal cell types were analyzed to identify commonly upregulated and downregulated genes under HG conditions. Five genes were commonly upregulated across all four cell types, while six genes were commonly upregulated in Ep, D, and S. Additionally, five genes were commonly upregulated in En, Ep, and S. Overall, 63 genes were upregulated in two or more cell types (Fig. 6A). These commonly upregulated genes were primarily enriched in GO terms related to inflammatory response, leukocyte chemotaxis, and innate immune response (Fig. 6B). Two genes were commonly downregulated across all four cell types. Twelve genes were commonly downregulated in Ep, D, and S, while one gene was commonly downregulated in En, D, and S. Additionally, two genes were commonly downregulated in En, D, and Ep, and two genes were commonly downregulated in En, S, and Ep. In total, 274 genes were downregulated in two or more cell types (Fig. 6C). These commonly downregulated genes were mainly enriched in GO terms related to modulation of chemical synaptic transmission, synapse organization, regulation of cytoskeleton organization, cell-cell adhesion, and extracellular matrix organization (Fig. 6D). We constructed a protein-protein interaction (PPI) network of the differentially expressed genes (DEGs) in these four cell types (Fig. S4) and identified the top-ranking hub genes, which were visualized in a heatmap (Fig. 6E). Notably, CCL5 appeared in both D and S. Constructing a PPI network of all hub genes from the four cell types revealed that CCL5 is a central hub gene, potentially related to all immune factors (Fig. 6F). We further validated the expression levels of these hub genes using qPCR. In D, genes related to inflammation and chemotaxis (IL6, CXCL8, CCL5) were upregulated, while genes associated with neurons (TUBB3), synapses, type I collagen (COL1A1), and cysteine protease inhibitors (CST1) were downregulated. In S, immune and chemotaxis-related genes (CCL5, IFIT3, CXCL10, TGM1, SERPINA1, IFIT1) were upregulated, and COL1A1 was downregulated. In Ep, immune-related factors (TGM1, SERPINA1) were upregulated, whereas genes related to cell proliferation and transcription factors (CCN2, ZFP36) were downregulated. In En, immune and chemotaxis-related genes (IFIT3, ISG15, IFIT1, CCL2) were upregulated (Fig. 6G). These results indicate that HG leads to the upregulation of inflammation and chemotaxis-related genes across all cell types, with CCL5 being a potentially critical factor.

### 3.5 LBGP mitigates HG-induced damage in the full-thickness biomimetic corneal model

**Fig. 7:**
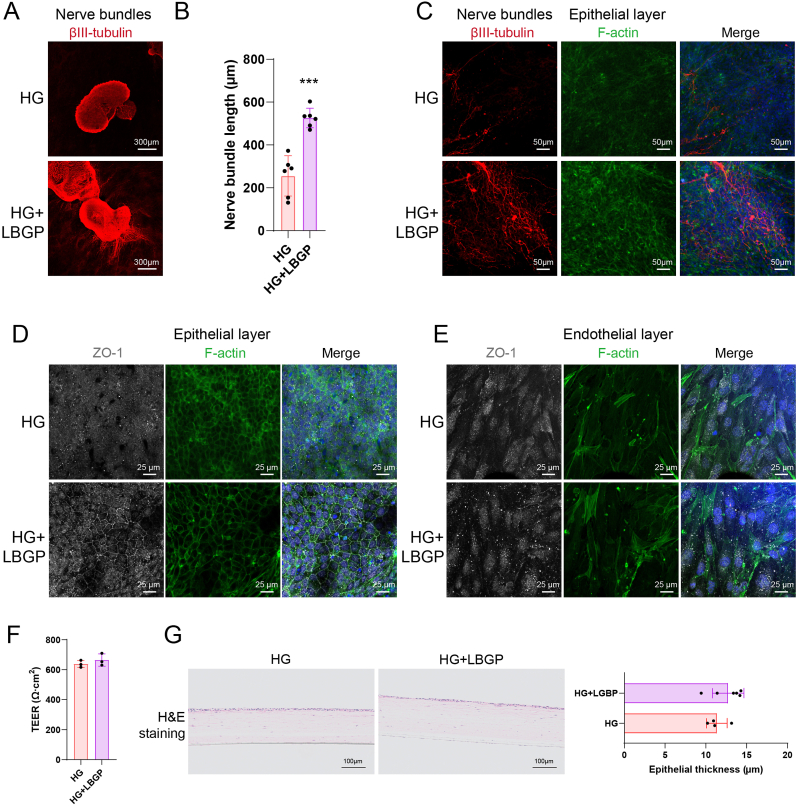
Protective Effects of LBGP Against HG-Induced Damage in the Full-Thickness Corneal Model. (A) Immunofluorescence staining of βIII-tubulin showing nerve bundles in HG and HG + LBGP groups. (B) Quantification of nerve bundle length in HG and HG + LBGP groups. (C) Immunofluorescence staining of βIII-tubulin (red) and F-actin (green) in the corneal epithelial layer. (D) Immunofluorescence staining of ZO-1 (white) and F-actin (green) in the corneal epithelial layer. (E) Immunofluorescence staining of ZO-1 (white) and F-actin (green) in the corneal endothelial layer. (F) Transepithelial electrical resistance (TEER) measurements comparing barrier integrity in HG and HG + LBGP groups. (G) Hematoxylin and eosin (H&E) staining of corneal epithelium, quantifying epithelial thickness. (∗∗∗, p < 0.001). (For interpretation of the references to colour in this figure legend, the reader is referred to the Web version of this article.)

The cytotoxicity of LBGP on corneal epithelial cells was evaluated under high-glucose conditions. It was found that LBGP promoted cell viability across a range of concentrations from 0.8 to 1000 μg/ml (Fig. S5A). Scratch wound assays demonstrated that LBGP notably enhanced corneal epithelial cell migration at a concentration of 100 μg/ml (Fig. S5B). Therefore, 100 μg/ml was selected as the working concentration for LBGP. LBGP was added along with HG to evaluate its protective effects against HG-induced damage. Immunofluorescence results showed that the addition of LBGP in the HG condition enhanced the growth of DRGO nerve bundles, resulting in a denser nerve network (Fig. 7A). The nerve bundle length was 526.1 ± 41.60 μm, significantly longer than the 255.7 ± 85.53 μm observed in the HG group (Fig. 7B). Local images revealed that, compared to the disintegration of nerves in the HG group, the nerve network in the HG + LBGP group was restored and capable of innervating corneal epithelial cells (Fig. 7C). Staining of the epithelial layer cells showed partial restoration of the tight junction marker ZO-1 in the HG + LBGP group, although not to the level of the NC group (Fig. 7D). Staining of the endothelial layer revealed that LBGP treatment did not restore the tight junctions of corneal endothelial cells, which retained a fibrotic shape similar to that observed in the HG group (Fig. 7E). Barrier function assessment showed that the TEER values for HG and HG + LBGP were 638.2 ± 19.19 Ω cm^2^ and 664.7 ± 33.31 Ω cm^2^, respectively, indicating that LBGP did not remarkably improve the corneal barrier function disrupted by HG (Fig. 7F). H&E staining of the *in vitro* model showed that the epithelial thickness in the HG + LBGP group was 12.74 ± 1.77 μm, which was not significantly different from the 11.35 ± 1.10 μm in the HG group (Fig. 7G). These results suggest that LBGP can partially restore the nerve and epithelial structure of the cornea.

**Fig. 8:**
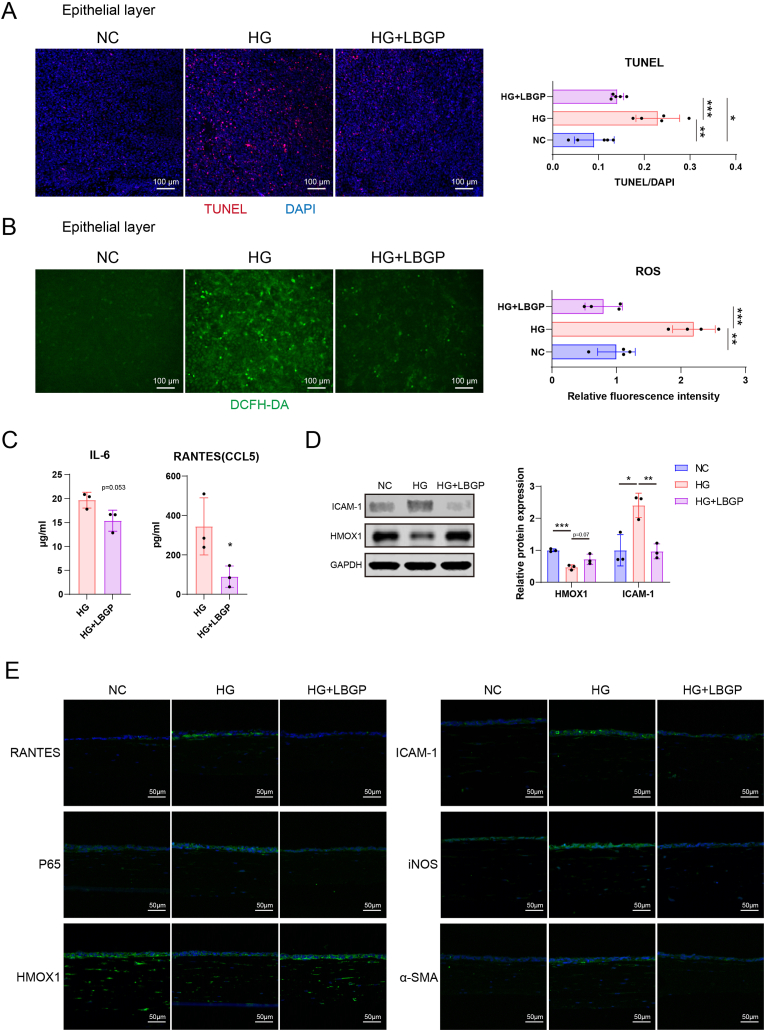
Effects of LBGP on Apoptosis, Oxidative Stress, and Inflammation in the HG-Treated Corneal Model. (A) TUNEL staining to detect apoptotic cells in normal control (NC), HG, and HG + LBGP groups. (B) DCFH-DA staining to detect reactive oxygen species (ROS) in NC, HG, and HG + LBGP groups. (C) ELISA measurements of IL-6 and RANTES (CCL5) levels in conditioned medium from HG and HG + LBGP groups. (D) Western blot analysis of ICAM-1 and HMOX1 protein expression in NC, HG, and HG + LBGP groups. (E) Immunofluorescence staining of RANTES, ICAM-1, P65, iNOS, HMOX1, and α-SMA in NC, HG, and HG + LBGP groups. (∗∗∗, p < 0.001; ∗∗, p < 0.01; ∗, p < 0.05).

Next, the effects of LBGP on HG-induced corneal inflammation and oxidative stress were examined. TUNEL staining showed that the percentages of TUNEL-positive corneal epithelial cells in the NC, HG, and HG + LBGP groups were 0.091 ± 0.039, 0.230 ± 0.043, and 0.141 ± 0.012, respectively. This indicates that HG induction increases the proportion of apoptotic cells while adding LBGP significantly reduces the apoptosis rate (Fig. 8A). Similar results were observed for ROS detection. The relative fluorescence intensity of DCFH-DA in corneal epithelial cells was 1.00 ± 0.25 in the NC group, 2.20 ± 0.29 in the HG group, and 0.80 ± 0.25 in the HG + LBGP group. This indicates that HG induction substantially increases intracellular ROS levels, while LBGP addition reduces ROS levels (Fig. 8B). ELISA was used to measure the changes in immune and chemotactic factors in the conditioned medium after LBGP treatment. The concentration of IL-6 after LBGP treatment was 15.36 ± 17.87 μg/ml, lower than the 19.66 ± 13.49 μg/ml in the HG group. The concentration of RANTES (CCL5) after LBGP treatment was 66.83 ± 44.33 μg/ml, notably lower than the 258.2 ± 118.1 μg/ml in the HG group (Fig. 8C). Western blot (WB) analysis of proteins extracted from all cells in the model showed that the immune-related protein ICAM-1 was upregulated in the HG group, and this upregulation was mitigated in the HG + LBGP group. The antioxidant protein HMOX1 was significantly downregulated after HG treatment, but LBGP promoted the upregulation of this protein under HG conditions, enhancing antioxidant capacity (Fig. 8D). Histological staining results showed elevated signals for the chemokine RANTES (CCL5), immune-related proteins ICAM-1 and P65, and the oxidative stress-related protein iNOS in the HG group, whereas these signals were reduced in the HG + LBGP group. The fibrotic marker α-SMA followed a similar trend. Conversely, the signal for the antioxidant protein HMOX1 was decreased in the HG group but increased after the addition of LBGP (Fig. 8E). These results indicate that LBGP can mitigate HG-induced oxidative stress and inflammatory response including CCL5.

### 3.6 LBGP alleviates corneal nerve damage, immune response, and oxidative stress in diabetic mice

**Fig. 9:**
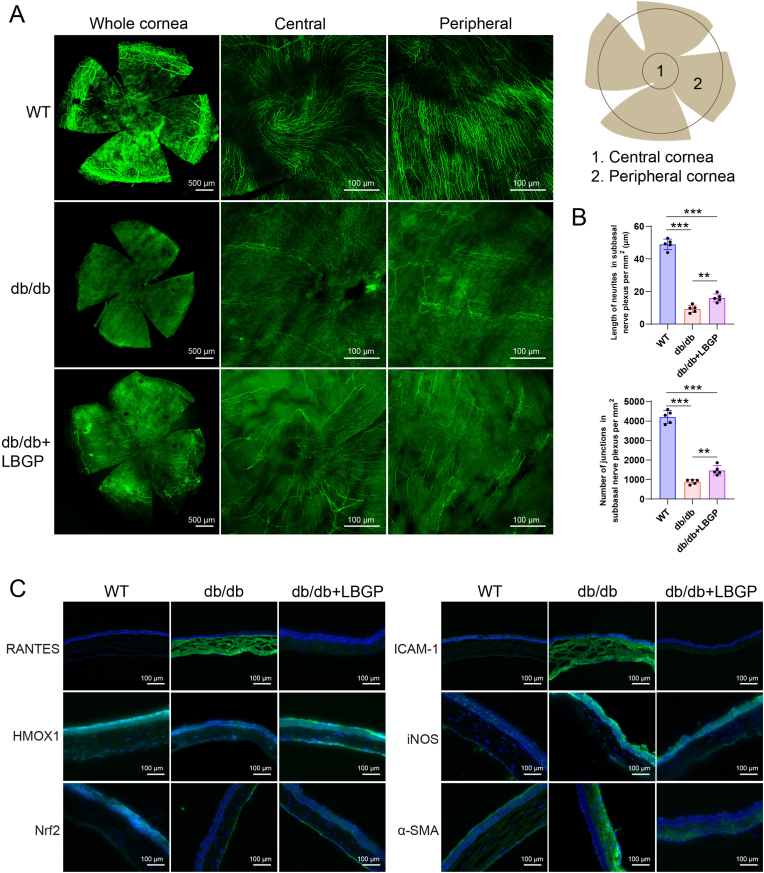
Alleviating Effects of LBGP on Corneal Nerve Regeneration, Inflammation, and Oxidative Stress in db/db Mouse Model. (A) βIII-tubulin immunostaining of whole cornea, central cornea, and peripheral cornea in wild-type (WT), db/db, and db/db + LBGP groups. (B) Quantification of the length of neurites in the subbasal nerve plexus per mm^2^ and the number of junctions in the subbasal nerve plexus per mm^2^. (C) Immunofluorescence staining of RANTES, HMOX1, Nrf2, ICAM-1, iNOS, and α-SMA in the corneas of WT, db/db, and db/db + LBGP groups. The scale bars represent 100 μm. (∗∗p < 0.01, ∗∗∗p < 0.001).

The db/db mice as a diabetic *in vivo* model were used to observe the therapeutic effects of LBGP on the cornea. Eight-week-old db/db mice were treated for three months. Post-treatment, blood glucose levels in the mice did not show significant changes (data not shown). Whole-mount corneal staining revealed that the subbasal nerve plexus, marked by βIII-tubulin, formed a physiological spiral structure in the central cornea of wild-type (WT) mice. However, this structure was almost entirely lost in db/db mice. In the db/db + LBGP group, partial restoration of the corneal nerve spiral structure was observed. Additionally, a dense nerve network was found in the peripheral cornea of WT mice, whereas this network disintegrated significantly in db/db mice. The db/db + LBGP group showed partial regeneration of the peripheral corneal subbasal nerve plexus, gradually innervating corneal epithelial cells (Fig. 9A). Morphological quantification of the subbasal nerve plexus *in vivo* models indicated that the length of neurites in the subbasal nerve plexus per mm^2^ was 48.96 ± 2.88 mm in the WT group, dramatically reduced to 9.31 ± 2.02 mm in db/db mice, and restored to 16.06 ± 2.22 mm after LBGP treatment. The number of junctions in the subbasal nerve plexus per mm^2^ was also assessed, with values of 4207 ± 293 in the WT group, 879 ± 103 in the db/db group, and 1468 ± 217 in the db/db + LBGP group, showing a similar trend (Fig. 9B). The corneas of the mice were fixed, cryosectioned, and subjected to immunohistochemical analysis. Chemokine RANTES (CCL5) and immune-related factor ICAM-1 were highly expressed in the corneal stroma of the db/db group, with reduced fluorescence signals after LBGP treatment. The antioxidant factor HMOX1 was highly expressed in the corneal epithelium of the WT group but showed weakened signals in the db/db group, which were enhanced again in the db/db + LBGP group. The oxidative stress marker iNOS showed opposite results, with the strongest signals in the db/db group and weaker signals in the WT and db/db + LBGP groups. The key antioxidant transcription factor Nrf2 was highly expressed in the epithelial layer of the WT group but showed weaker signals in the db/db and db/db + LBGP groups. The fibrosis marker α-SMA was highly expressed in the stroma of the db/db group, and this trend was mitigated in the db/db + LBGP group (Fig. 9C). These results indicate that in the db/db mouse model, corneal nerves are damaged, with increased oxidative stress, immune responses, and fibrosis, alongside decreased antioxidant capacity. After LBGP administration, these trends were effectively reversed.

## 4 Discussion

Diabetic keratopathy (DK) is a significant complication of diabetes mellitus, resulting in corneal damage and vision impairment. Developing an *in vitro* full-thickness corneal model is essential for understanding the pathophysiology of DK and evaluating potential therapeutic agents. This study is the first to construct a full-thickness biomimetic corneal model containing corneal epithelium, nerves, stroma, and endothelium, which was used to assess the protective effects of Lycium barbarum glycopeptide (LBGP) against high-glucose (HG)-induced corneal damage.

The cornea consists of both cellular and non-cellular components. In this study's *in vitro* model, various human corneal cell types were incorporated along with collagen, representing the non-cellular component. This comprehensive model replicates the complex structure of the human cornea and integrates corneal nerves, a crucial enhancement often missing in previous models, allowing for a more accurate simulation of corneal physiology and pathology. Constructing an *in vitro* full-thickness corneal model is vital for several reasons. Firstly, it more accurately replicates the complex structure and function of the human cornea compared to traditional models that typically focus on individual corneal cell types, reducing the use of experimental animals [36]. This model provides a holistic view of corneal biology by including epithelial, nerve, stromal, and endothelial components, enabling the study of cellular interactions and responses to various conditions. In the context of diabetic keratopathy (DK), such a model is particularly valuable. DK is characterized by various pathological changes, including reduced corneal nerve density and sensitivity, epithelial defects, delayed wound healing, and increased inflammation and oxidative stress [3]. Traditional models have limitations in replicating these multifaceted changes, hindering the ability to study the disease in detail and develop effective treatments. This study's *in vitro* full-thickness corneal model overcomes these limitations by providing a more physiologically relevant platform. Moreover, in the current study, the model's ability to evaluate the protective effects of LBGP against HG-induced damage highlights its potential for screening therapeutic agents. The model provides a controlled environment to test these drugs before moving to more complex *in vivo* studies.

This study utilized a high-glucose environment to simulate the pathogenesis of DK in an *in vitro* model. High glucose leads to the disintegration of corneal nerve bundles. Compared to the NC group, the HG group exhibited significant fragmentation and reduced length of nerve bundles, a hallmark of DK that results in decreased corneal sensitivity and impaired wound healing. RNA-seq analysis also revealed that in the HG group, genes related to axon and synapse formation were significantly downregulated, while genes associated with inflammatory responses were upregulated. Corneal nerves are crucial in corneal immune crosstalk, with damaged nerve axons potentially inducing immune cell activity directly or indirectly through the release of neuropeptides or other cytokines [37]. After the addition of LBGP, the length and density of nerve bundles were enhanced in the LBGP-treated group compared to the HG group. This indicates that LBGP possesses substantial neuroprotective properties, helping to restore nerve integrity compromised by high glucose. In the diabetic *in vivo* model, LBGP treatment for three months led to partial restoration of the physiological spiral structure of the subbasal nerve plexus in the central cornea, which was almost completely lost in untreated db/db mice. Additionally, the peripheral corneal nerve network, which had significantly disintegrated in db/db mice, showed partial regeneration in the LBGP-treated group, indicating enhanced nerve regeneration and improved corneal innervation. This is particularly important given that corneal nerve damage is a major complication of DK, leading to decreased corneal sensitivity and impaired wound healing [38]. In diabetic patients, the density of the subbasal nerve plexus is significantly reduced, nerve branching decreases, and nerve tortuosity increases [39,40]. These changes contribute to a decrease in corneal sensation [41]. Previous studies have shown that various drugs, such as nerve growth factor (NGF), have neuroprotective properties in promoting nerve regeneration and preventing nerve damage in diabetic models [42,43]. However, besides their relatively high cost of administration [44], these drugs often cannot simultaneously alleviate both inflammation and oxidative stress. Previous studies have shown that Lycium barbarum extracts, such as Lycium barbarum polysaccharides (LBP), possess neuroprotective and regenerative properties. Zhao et al. found that the antioxidant mechanisms of LBP promote the repair and regeneration of cavernous nerves following injury [45]. LBP has also been shown to protect the optic nerve, as reported by studies indicating that LBP promotes the regeneration and survival of retinal ganglion cell (RGC) axons following optic nerve crush (ONC) [46]. Lycium barbarum glycopeptide (LBGP), a further purified active component of LBP, has also been reported to be effective in models of retinal diseases. Kong et al. demonstrated that oral LBGP treatment in a mouse model of retinal photoreceptor damage improved photoreceptor survival, retinal light response, and visual behavior while inhibiting microglial activation and reducing pro-inflammatory cytokine expression [27]. Additionally, LBGP has been shown to prevent stress-induced anxiety by modulating oxidative stress and ferroptosis in the medial prefrontal cortex, likely through the activation of the transcription factor Nrf2 and subsequent upregulation of antioxidant defenses [29,47]. Lakshmanan et al. administered LBGP to a mouse model of retinal ganglion cell (RGC) injury and found that LBGP treatment significantly promoted the recovery of retinal blood flow, preserved RGC density, and protected retinal function [48]. These studies collectively highlight the neuroprotective and reparative effects of LBGP on corneal nerve damage from diabetic high-glucose conditions.

A high-glucose (HG) environment also induces damage to other corneal cells. In the *in vitro* model used in this study, the integrity of both the corneal epithelial and endothelial cell layers was compromised, with a reduction in epithelial thickness and damage to epithelial microvilli. The barrier function in the HG group was significantly reduced, with tight junctions and cellular junctions in both the epithelial and endothelial layers being disrupted. This finding is consistent with studies showing that the connections between corneal epithelial cells are affected in diabetic keratopathy patients [49]. Additionally, autophagy was observed in corneal stromal cells under HG conditions. Inflammation and oxidative stress markers were significantly elevated in corneal cells exposed to HG, with an increased rate of apoptosis and elevated ROS levels leading to oxidative damage. Pro-inflammatory cytokines, including IL-6 and RANTES, were significantly upregulated in the HG group, reflecting an enhanced inflammatory response. Clinically, DK patients typically exhibit elevated levels of ROS and immune factors in epithelial samples and tear fluid, which our model successfully replicated [4,50]. The transcriptomic results revealed that in the HG group, many genes associated with inflammatory responses, oxidative stress, and apoptosis were upregulated. LBGP treatment also improved the integrity of epithelial cells. Immunofluorescence staining for ZO-1 indicated that tight junctions in the LBGP-treated group were partially restored, although not to the level of the normal control group. Furthermore, multiple findings in this study demonstrated that LBGP significantly reduced markers of inflammation, oxidative stress and apoptosis in the corneal model exposed to high glucose. In the db/db mouse model, signals related to oxidative stress, immune responses, and fibrosis were elevated, while antioxidant signals were downregulated. However, after three months of LBGP administration, these trends were reversed, indicating the protective effects of LBGP in modulating these damaging processes. The observed therapeutic effects suggest that LBGP acts directly on corneal cells to exert its protective effects. Potential mechanisms include the activation of antioxidant pathways, inhibition of pro-inflammatory cytokine production, and promotion of cellular repair and regeneration processes. Increasing evidence supports the role of Lycium barbarum extracts (LBP, LBGP) in anti-inflammation, antioxidation, and antifibrosis. Their anti-inflammatory effects may be mediated through the inhibition of the NF-κB pathway [28]. LBP not only exhibits anti-scar properties in the cornea [51,52] but also promotes corneal re-epithelialization after alkali burns [53,54]. Research has also shown that LBP protects rat corneal epithelial cells from UV-induced apoptosis by attenuating the mitochondrial pathway and inhibiting JNK phosphorylation [55]. Moreover, Xie et al. reported that LBGP accelerated corneal epithelial recovery in a rat model of mechanical injury by enhancing epithelial cell proliferation and inhibiting apoptosis [56]. Collectively, these findings, along with our study, demonstrate that LBGP has significant therapeutic potential for treating nerve damage and related diseases characterized by inflammation and oxidative stress, making it a promising candidate for future drug development. Additionally, the multifaceted actions of LBGP provide a comprehensive therapeutic approach, which may offer greater overall benefits in managing DK compared to drugs that target only one aspect of the disease.

C-C motif chemokine ligand 5 (CCL5), also known as RANTES, is a chemokine involved in regulating immune responses by attracting leukocytes to sites of inflammation. CCL5 interacts with several chemokine receptors, such as CCR1, CCR3, and CCR5, to mediate its effects, extending to various pathological conditions, including inflammatory diseases, cancer, and metabolic disorders like diabetes [57]. In this study, CCL5 emerged as a critical factor, significantly upregulated in the HG group *in vitro* model and highly expressed in the corneas of db/db mice. CCL5 expression is markedly elevated, particularly in the corneal epithelium and stroma. RNA-seq results identified CCL5 as the most crucial hub gene. After the addition of LBGP to the HG-induced *in vitro* model, the concentration of RANTES (CCL5) in the conditioned medium decreased. Similarly, the signal for RANTES was also reduced in the corneas of LBGP-treated db/db mice. CCL5 is upregulated in diabetes, contributing to the chronic inflammatory state associated with the disease. Elevated levels of CCL5 have been found in the serum and tissues of diabetic patients [58,59], indicating its significant role in mediating diabetes-related inflammation. CCL5 recruits immune cells such as T cells, monocytes, and macrophages to sites of inflammation, exacerbating tissue inflammation and promoting the development of diabetic complications, including nephropathy [60] and retinopathy [59]. By binding to its receptors, CCL5 activates signaling pathways that promote the production of pro-inflammatory cytokines and chemokines, further amplifying the inflammatory response [61]. Although there have been no studies on CCL5 promoting DK, there have been studies on corneal injury associated with CCL5. Eslani et al. reported that challenging wounded corneal epithelial cells with ultrapure lipopolysaccharide (LPS) resulted in an increase in IL-6, TNF-a, and CXCL8/IL8 and CCL5/RANTES [62]. CCL5 promotes corneal inflammation by attracting macrophages and T cells to the corneal tissue [63]. The resulting chronic inflammation impairs corneal wound healing and regeneration, worsening the clinical manifestations of DK, such as delayed epithelial healing, increased corneal haze, and reduced corneal sensitivity [64]. Therefore, in the current study, addressing the upregulation of CCL5 in diabetic conditions could provide a new therapeutic avenue to reduce inflammation, protect corneal nerves, and promote wound healing in diabetic keratopathy and other related diseases.

However, this study has some limitations. Although the *in vitro* model includes the major structures and cell types of the cornea, it may not fully replicate the *in vivo* corneal environment, particularly the interactions with the tear film and eyelid mechanics. The spatial distribution of relevant protein expression in the corneas between the high-glucose group of the *in vitro* model and db/db mice also displayed some degree of inconsistency, which may be attributed to differences in the model induction time. The *in vitro* model has a shorter induction time compared to the chronic diabetic model *in vivo*, making it more akin to an acute model. Additionally, the positioning of the corneal nerves differs from that of the natural cornea, lacking the simulation of limbal nerve positioning. Future research will use cornea-on-a-chip technology to address these limitations. The cornea-on-a-chip can confine different cells to designated areas, simulate a corneal model with physiological structure [65], and replicate the cornea's stimulation by aqueous humor and tear fluid [66]. As a supplement to animal research, this model has the potential to accelerate translational studies, particularly the preclinical screening of ophthalmic drugs, thus advancing the clinical treatment of corneal diseases. The biomimetic full-thickness corneal model developed in this study provides a valuable platform for exploring a wide range of potential therapeutic agents beyond LBGP. Future research could utilize this model to study other corneal diseases and conditions, such as dry eye syndrome, corneal dystrophies, and postoperative healing. Additionally, the inclusion of patient-derived cells could facilitate the investigation of personalized treatment regimens tailored to individual patient conditions, potentially leading to more effective and customized therapeutic interventions.

In conclusion, this study successfully developed and validated a biomimetic full-thickness corneal model that replicates the significant structures of the human cornea, including epithelial cells, stromal cells, endothelial cells, and nerves. By exposing the model to a high-glucose (HG) environment, the study effectively simulated the morphological and functional changes characteristic of diabetic keratopathy (DK), including inflammation, oxidative stress, and nerve damage. The therapeutic potential of Lycium barbarum glycopeptide (LBGP) was demonstrated, as it alleviated HG-induced corneal damage, promoted nerve regeneration, and reduced inflammation and oxidative stress in both *in vitro* and *in vivo* models. This biomimetic model represents a significant advancement in corneal research, providing a robust and versatile platform for studying the pathogenesis of DK and other corneal disorders under controlled conditions. Furthermore, identifying LBGP as a promising therapeutic agent offers a new avenue for developing effective treatments for DK. These findings enhance our understanding of corneal disease mechanisms and open up opportunities for future translational research and clinical applications in ophthalmology.

## CRediT authorship contribution statement

**Zekai Cui:** Writing – original draft, Visualization, Validation, Software, Resources, Methodology, Investigation, Funding acquisition, Conceptualization. **Xiaoxue Li:** Validation, Resources, Methodology, Investigation. **Yiwen Ou:** Resources, Methodology. **Xihao Sun:** Resources, Methodology. **Jianing Gu:** Methodology. **Chengcheng Ding:** Methodology, Funding acquisition. **Zhexiong Yu:** Resources. **Yonglong Guo:** Methodology, Investigation. **Yuqin Liang:** Methodology. **Shengru Mao:** Methodology. **Jacey Hongjie Ma:** Resources. **Hon Fai Chan:** Resources, Conceptualization. **Shibo Tang:** Supervision, Project administration, Conceptualization. **Jiansu Chen:** Writing – review & editing, Supervision, Project administration, Investigation, Funding acquisition, Conceptualization.

## Declaration of generative AI and AI-assisted technologies in the writing process

During the preparation of this work the authors used ChatGPT-4o in order to polish the manuscript. After using this tool, the authors reviewed and edited the content as needed and take full responsibility for the content of the publication.

## Declaration of competing interest

The authors declare the following financial interests/personal relationships which may be considered as potential competing interests:Zekai Cui, Xihao Sun, Jiansu Chen has patent #CN202210826736.9 pending to Aier Eye Hospital Group Co., Ltd. Zekai Cui, Xiaoxue Li, Jiansu Chen has patent #CN202311810762.3 pending to Aier Eye Hospital Group Co.,Ltd. Z.Y. was employed by company Tianren Goji Biotechnology Co., Ltd. If there are other authors, they declare that they have no known competing financial interests or personal relationships that could have appeared to influence the work reported in this paper.

## Data Availability

Data will be made available on request.
