# Supplementary material for: Novel full-thickness biomimetic corneal model for studying pathogenesis and treatment of diabetic keratopathy
Source: Mater Today Bio. 2024 Dec 16;30:101409. doi: 10.1016/j.mtbio.2024.101409 (PMC11729032; doi:10.1016/j.mtbio.2024.101409)
Supplement: Multimedia component 1 [file mmc1.pdf]

Supplementary file

Figure S1. Design schematic of the DRGO culture mold.

Figure S2. Preprocessing results of the cytokine microarray. (A) PCA results showing intra-group and inter-group differences between HG and NC groups. (B) Volcano plot displaying differentially expressed cytokines.

Figure S3. Preprocessing results of RNA-seq. (A) PCA results showing intra-group and inter-group differences among D, S, Ep, and En cells in NC and HG conditions. (B) Volcano plot displaying differentially expressed genes in these four cell types.

Figure S4. PPI network of differentially expressed genes in D, S, Ep, and En cells under NC and HG conditions. The red markers indicate the hub genes for each cell type.

Figure S5. LBGP cytotoxicity and cell viability assessment. (A) CCK8 results showing the effects of LBGP (0.8-1000 μg/ml) on CEpCs viability under HG conditions. (B) Scratch assay demonstrating the impact of LBGP (20 and 100 μg/ml) on CEpCs migration ability under HG conditions. (*, $p<0.05$; **, $p<0.01$)

Table S1. qPCR primer information.

Table S2. Detailed data from the cytokine microarray (EXCEL file).

Table S3. Detailed RNA-seq analysis data (EXCEL file).

Table S4. Venn diagrams and PPI analysis results of the four cell types (EXCEL file).

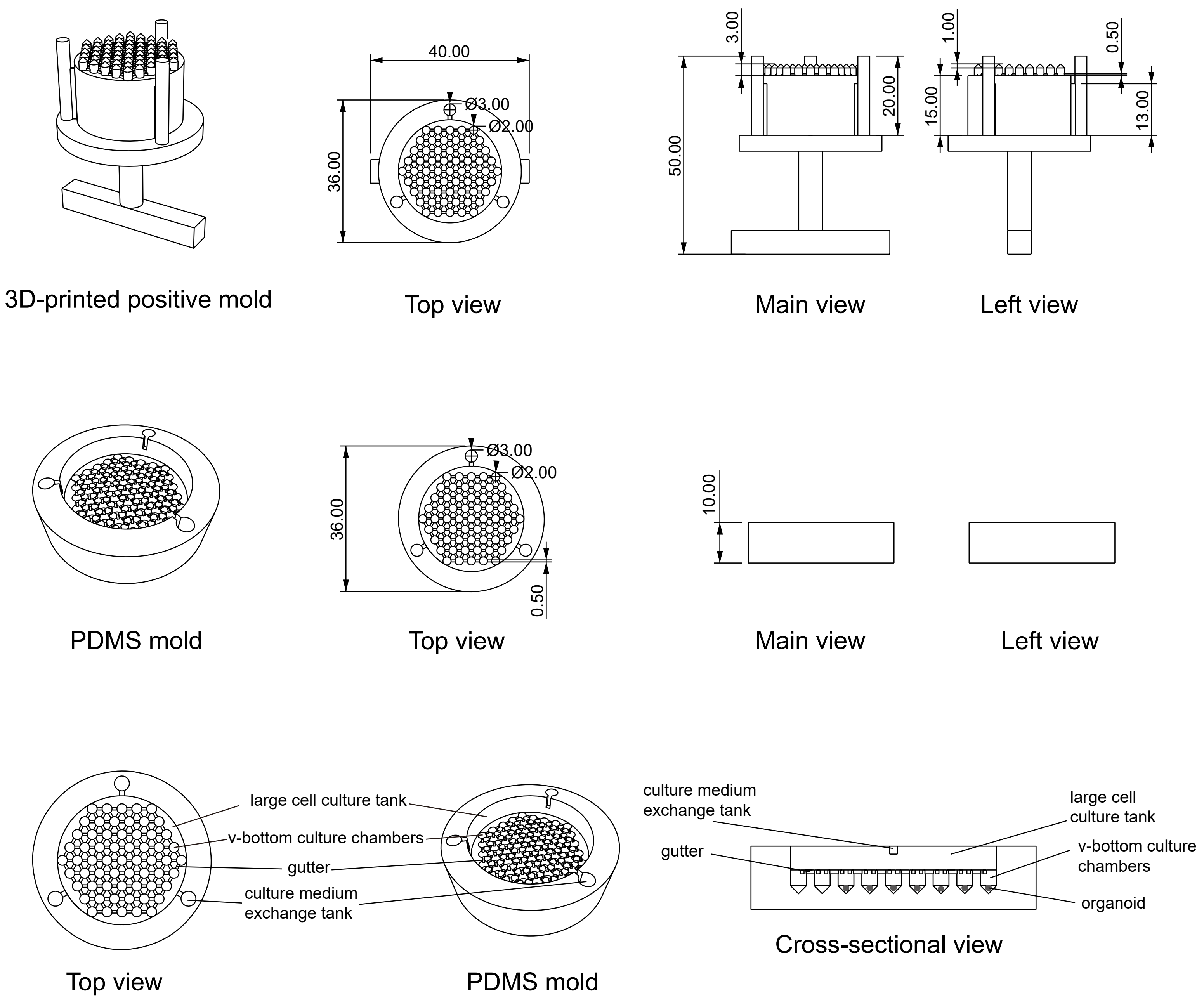

Figure S1. Design schematic of the DRGO culture mold.

Cytokine microarray

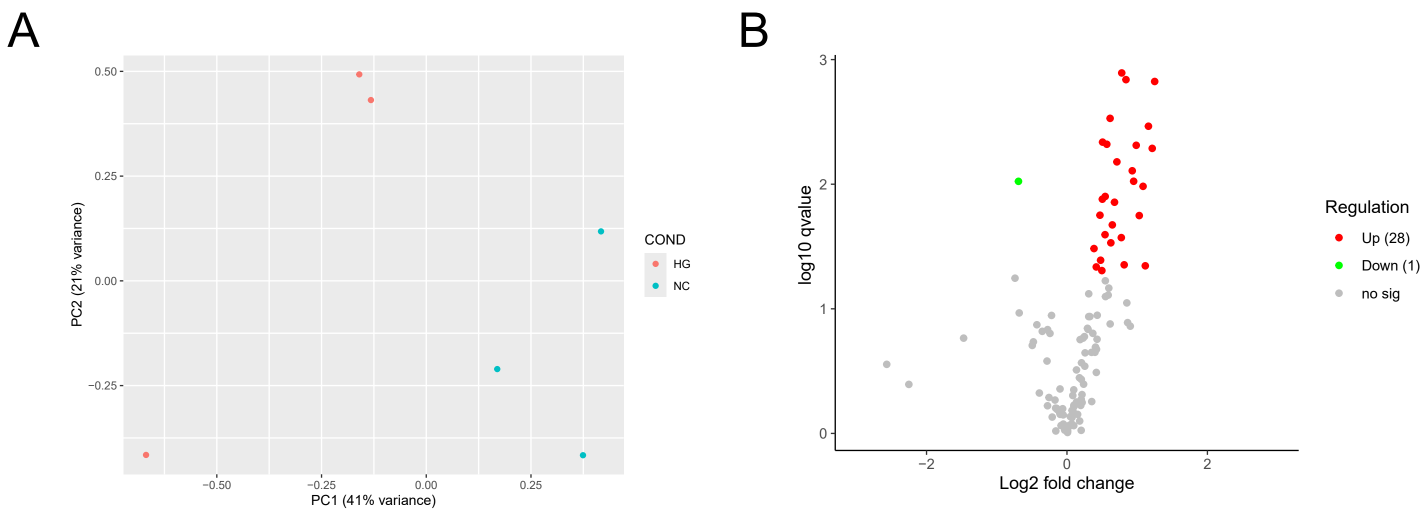

Figure S2. Preprocessing results of the cytokine microarray. (A) PCA results showing intra-group and inter-group differences between HG and NC groups. (B) Volcano plot displaying differentially expressed cytokines.

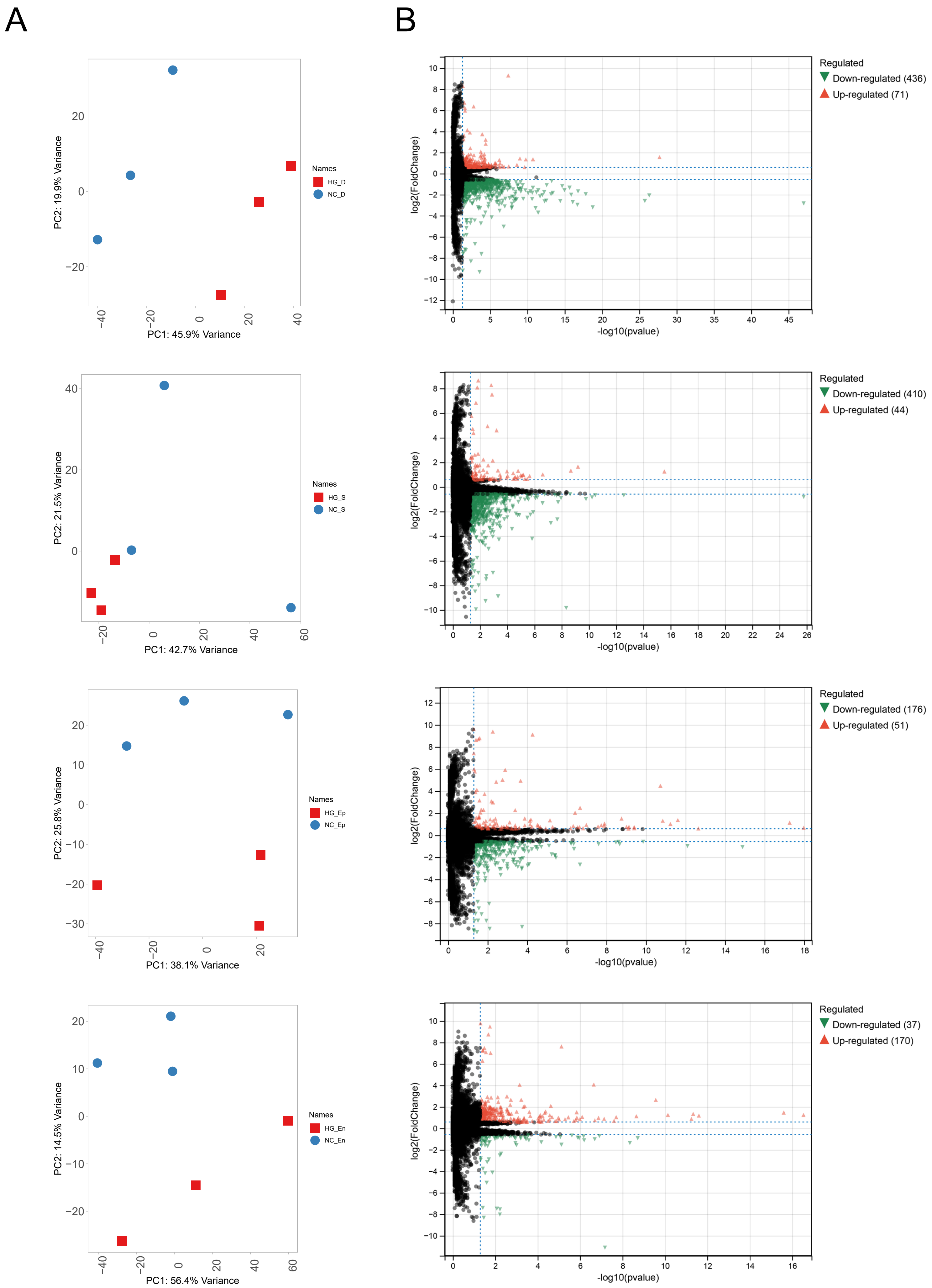

Figure S3. Preprocessing results of RNA-seq. (A) PCA results showing intra-group and inter-group differences among D, S, Ep, and En cells in NC and HG conditions. (B) Volcano plot displaying differentially expressed genes in these four cell types.

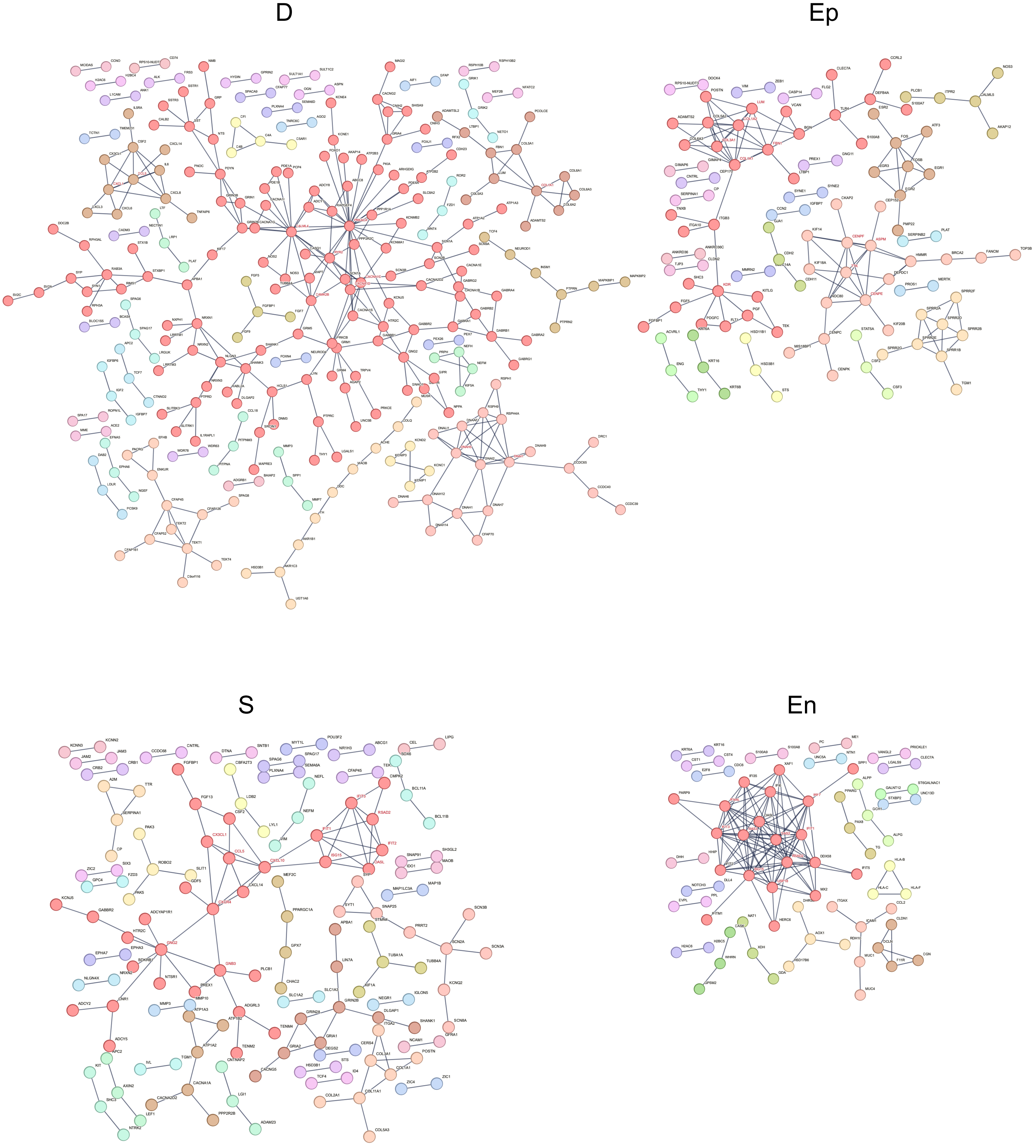

Figure S4. PPI network of differentially expressed genes in D, S, Ep, and En cells under NC and HG conditions. The red markers indicate the hub genes for each cell type.

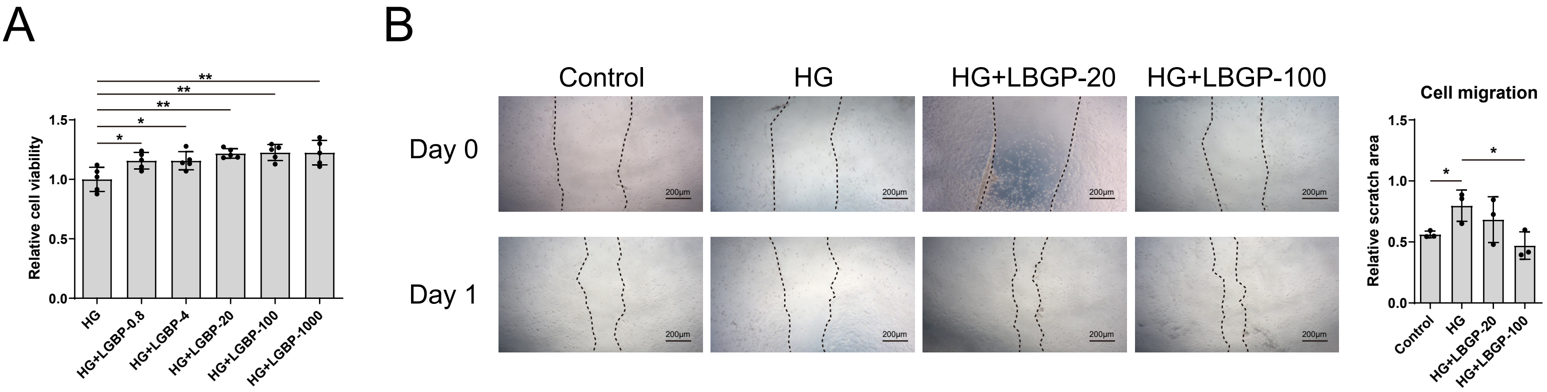

Figure S5. LBGP cytotoxicity and cell viability assessment. (A) CCK8 results showing the effects of LBGP (0.8-1000 μg/ml) on CEpCs viability under HG conditions. (B) Scratch assay demonstrating the impact of LBGP (20 and 100 μg/ml) on CEpCs migration ability under HG conditions. (*, p<0.05; **, p<0.01)

Table S1. qPCR primer information.

Gene name	Accession number	Sequence (5'->3')	Product length
IL6	NM_000600.5	F: ACCCCCAGGAGAAGATTCCA<br>R: TCACCAGGCAAGTCTCCTCA	251
COL1A1	NM_000088.4	F: AGTGGTTTGGATGGTGCCAA<br>R: GCACCATCATTTCCACGAGC	170
CXCL8	NM_000584.4	F: TCTGCAGCTCTGTGTGAAGG<br>R: TTCTCAGCCCTCTTCAAAAACT	248
TUBB3	NM_006086.4	F: GGAGATCGTGCACATCCAGG<br>R: CAGGCAGTCGCAGTTTTCAC	385
CCL5	NM_002985.3	F: GGATCAAGACAGCACGTGGA<br>R: TCGGGTGACAAAGACGACTG	248
GABBR1	NM_001470.4	F: CAACGCCACCTCAGAAGGTT<br>R: GGAAACAGTGCCCCGATGTA	462
CST1	NM_001898.3	F: TGATGAGTGGGTACAGCGTG<br>R: GTCTGTTGCCTGGCTCTTAGT	111
IFIT3	NM_001549.6	F: GAGGGCAGTCATGAGTGAGG<br>R: GGCGTAGTTTCCCCAAGTGA	313
CXCL10	NM_001565.4	F: ACTGCCATTCTGATTTGCTGC<br>R: TGATGGCCTTCGATTCTGGA	238
TGM1	NM_000359.3	F: GAAATGCGGCAGATGACGAC<br>R: TCGGGGTTGTTTCCGATGAG	361
SERPINA1	NM_000295.5	F: GACACCGAAGAGGCCAAGAA<br>R: GCTGGCAGACCTTCTGTCTT	411
IFIT1	NM_001548.5	F: ATTTACAGCAACCATGAGTACAAA<br>R: GGCTTCCTCATTCTGGCCTT	226
ZFP36	NM_003407.5	F: GAAGGGCCACTCCTATCAGC<br>R: CCCCAAGAACCTCGGAAGAC	455
CCN2	NM_001901.4	F: AGAGCAGCTGCAAGTACCAG<br>R: GGCTCTGCTTCTCTAGCCTG	354
ISG15	NM_005101.4	F: GTGGACAAATGCGACGAACC<br>R: CTTGATCCTGCTCGGATGCT	307
CCL2	NM_002982.4	F: CTCTCGCCTCCAGCATGAAA<br>R: GGTGTCTGGGGAAAGCTAGG	378
GAPDH	NM_002046.6	F: TTCTTTTGCGTCGCCAGCC<br>R: TCCCGTTCTCAGCCTTGACG	236

Species: *homo sapiens*
